# Genome survey of pistachio (*Pistacia vera* L.) by next generation sequencing: Development of novel SSR markers and genetic diversity in *Pistacia* species

**DOI:** 10.1186/s12864-016-3359-x

**Published:** 2016-12-07

**Authors:** Elmira Ziya Motalebipour, Salih Kafkas, Mortaza Khodaeiaminjan, Nergiz Çoban, Hatice Gözel

**Affiliations:** 1 Department of Horticulture, Faculty of Agriculture, University of Çukurova, 01330 Adana, Turkey; 2 Pistachio Research Institute, Gaziantep, Turkey

**Keywords:** Pistachio, Genome survey, Genome size, SSR, Genetic diversity

## Abstract

**Background:**

Pistachio (*Pistacia vera* L.) is one of the most important nut crops in the world. There are about 11 wild species in the genus *Pistacia,* and they have importance as rootstock seed sources for cultivated *P. vera* and forest trees. Published information on the pistachio genome is limited. Therefore, a genome survey is necessary to obtain knowledge on the genome structure of pistachio by next generation sequencing. Simple sequence repeat (SSR) markers are useful tools for germplasm characterization, genetic diversity analysis, and genetic linkage mapping, and may help to elucidate genetic relationships among pistachio cultivars and species.

**Results:**

To explore the genome structure of pistachio, a genome survey was performed using the Illumina platform at approximately 40× coverage depth in the *P. vera* cv. Siirt. The K-mer analysis indicated that pistachio has a genome that is about 600 Mb in size and is highly heterozygous. The assembly of 26.77 Gb Illumina data produced 27,069 scaffolds at N50 = 3.4 kb with a total of 513.5 Mb. A total of 59,280 SSR motifs were detected with a frequency of 8.67 kb. A total of 206 SSRs were used to characterize 24 *P. vera* cultivars and 20 wild *Pistacia* genotypes (four genotypes from each five wild *Pistacia* species) belonging to *P. atlantica, P. integerrima, P. chinenesis, P. terebinthus,* and *P. lentiscus* genotypes. Overall 135 SSR loci amplified in all 44 cultivars and genotypes, 41 were polymorphic in six *Pistacia* species. The novel SSR loci developed from cultivated pistachio were highly transferable to wild *Pistacia* species.

**Conclusions:**

The results from a genome survey of pistachio suggest that the genome size of pistachio is about 600 Mb with a high heterozygosity rate. This information will help to design whole genome sequencing strategies for pistachio. The newly developed novel polymorphic SSRs in this study may help germplasm characterization, genetic diversity, and genetic linkage mapping studies in the genus *Pistacia*.

**Electronic supplementary material:**

The online version of this article (doi:10.1186/s12864-016-3359-x) contains supplementary material, which is available to authorized users.

## Background

*Pistacia* is a genus of flowering plants in the cashew family, Anacardiaceae, which also contains mango, poison ivy, poison oak, pepper tree, and sumac plants [1]. The *Pistacia* genus consists of at least 11 species and is estimated to be about 80 million years old [2]. The pistachio is native to the arid zones of Central Asia; it has been cultivated for 3000–4000 years in Iran and was introduced into Mediterranean Europe by the Romans at the beginning of the Christian era [3]. Pistachio cultivation extended westward from its center of origin to Italy, Spain, and other Mediterranean regions of Southern Europe, North Africa, and the Middle East, as well as to China and more recently to the United States and Australia [4, 5].

Currently, Iran, the United States, Turkey, and Syria are the main pistachio producers in the world [6]. The main cultivation area in Turkey is in the South East, especially in Gaziantep, Şanlıurfa, Siirt, and Kahramanmaraş provinces, which cover 90–95% of pistachio production. Moreover, wild trees of *Pistacia* species such as *P. atlantica* Desf., *P. eurycarpa*, Yalt., *P. lentiscus* L., and *P. terebinthus* L*.*, expanded in almost all parts of Anatolia. Other well-known *Pistacia* species in the world are *P. integerrima* Stewart and *P. chinensis* Bunge. [7]. Pistachio plants are long-living with a juvenile period of approximately 5–10 years. In addition, wild *Pistacia* species have edible seeds and are used as rootstock seed sources for cultivated *P. vera*, and sometimes, fruit consumption, oil extraction, soap production, and as forest trees [8].

Several molecular markers such as randomly amplified polymorphic DNA (RAPD) [9, 10], simple sequence repeat (SSR) [11], sequence-related amplified polymorphism (SRAP) [12], amplified fragment length polymorphism (AFLP) [1, 13], inter-simple sequence repeats (ISSR) [14], selectively amplified microsatellite polymorphic loci (SAMPL) [15], and single nucleotide polymorphism (SNP) [16] have been used to assess the genetic diversity, fingerprinting, phylogenetic relationships, germplasm characterization, sex determination, and genetic linkage mapping in cultivated and wild *Pistacia* species.

SSRs are useful tools as molecular markers and are very polymorphic due to their high mutation rate, which affects the number of repeat units [17]. They are very useful for assaying diversity in natural populations or germplasm collections, and for fingerprinting and parental identification. They are very valuable markers especially for genetic linkage mapping and evolutionary studies [18] and have a high level of transferability between closely related species. The development of SSR markers from *P. vera* [11, 19–21] and wild *Pistacia* species has been described in several studies [22, 23].

Next generation sequencing (NGS) has provided a new perspective for research, owing to its high throughout and speed of data generation. So far, NGS has been applied to genomics-based strategies to discover sequences for new SSR markers in plants, in a time and cost-effective manner [24]. SSR development studies from a genome survey were performed in different plant species [25–27]. Genome survey studies also provide information about genome structure of a plant species, including estimates of genome size, levels of heterozygosity, and repeat contents. A study by Horjales et al. [28] is the only one in the literature to estimate genome size in the genus *Pistacia.* The genome size of *P. terebinthus* was estimated to be 2C = 1.32 Gb by flow cytometry.

Recently, genetic structure analyses have focused on the collection, protection, and utilization of germplasm for a plant species [29, 30]. It is important to explore population structure to avoid false genetic trends and to identify cultivars with specific or minor alleles that will be important for molecular breeding programs [31]. However, as far as we know, information on the population structure of *Pistacia* collections assessed using a large and comprehensive set of SSR markers is limited.

In this study, we aim to (1) estimate the genome size, GC content, and heterozygosity rates of pistachio (*P. vera* cv. Siirt) using a genome survey, (2) to perform genome-wide characterization of SSRs in the *P. vera* genome, (3) develop novel SSR markers for *Pistacia* species from a genome survey study, (4) determine transferable and polymorphic SSR markers for other *Pistacia* species, and (5) reveal the population structure of *Pistacia* germplasm. To our knowledge, this is the first report revealing genome structure and genome-wide SSRs in pistachio. The results of this study will provide essential information for further studies in pistachio such as whole genome sequencing and SSR-based genetic linkage mapping.

## Results

### K-mer analysis

**Fig. 1:**
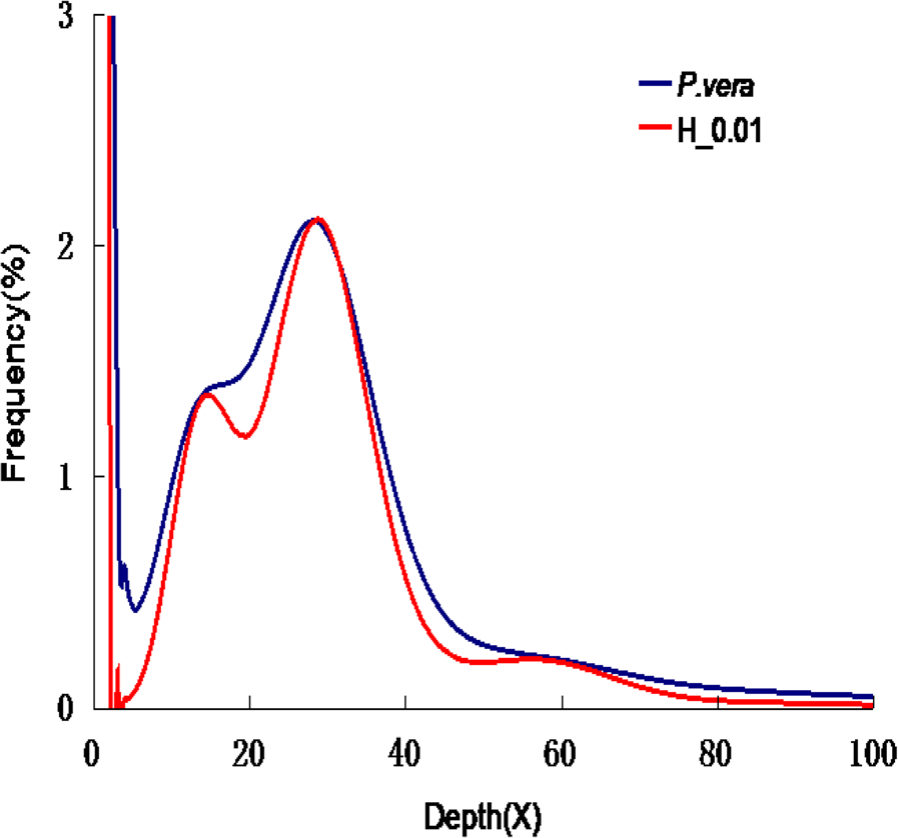
17 K-mer analysis for estimating the genome size of *Pistacia vera* cv. Siirt. The X-axis is depth (X) and the Y-axis is the proportion that represents the frequency at that depth. The H_0.01 means that the heterozygous rate is 1%

**Fig. 2:**
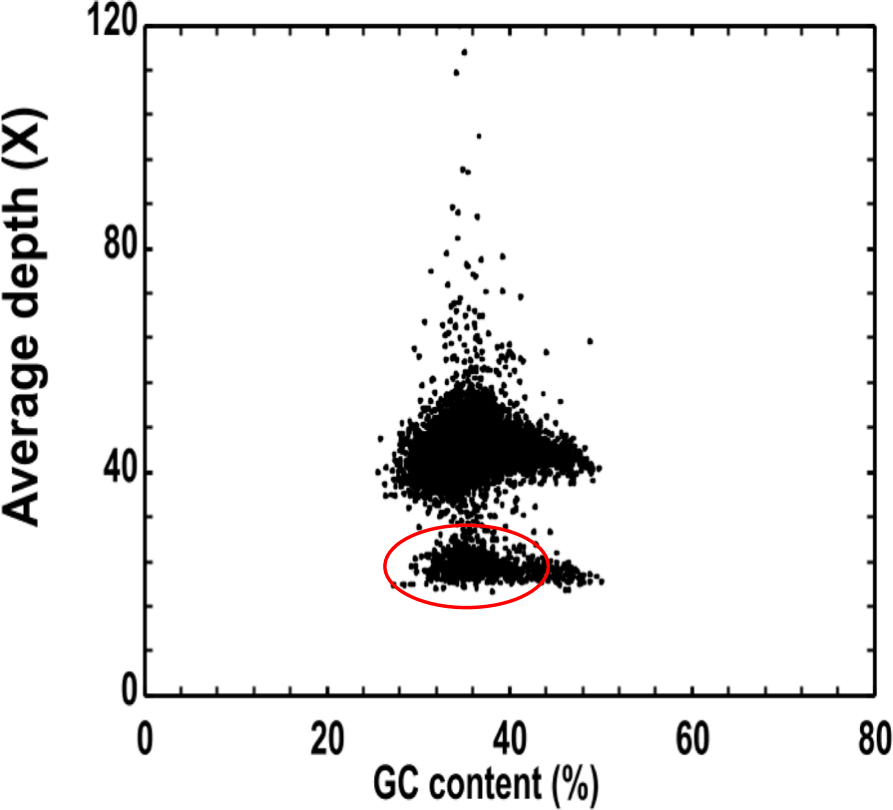
GC content and average sequencing depth. The X-axis represents GC content and the Y-axis represents the average depth. Red region whose average depth is around the half of main average depth, which may be caused by the high heterozygous rate

A total of 26.77 Gb were used for K-mer analysis. The 17-mer frequency distribution derived from the sequencing reads was plotted in Fig. 1; the peak of the 17-mer distribution was about 28, and the total K-mer count was 16,684,162,450; therefore, the genome size of pistachio was estimated as 596 Mb. A small peak observed at half the peak-depth showed a high level of heterozygosity for *P. vera*. Simulation of the *P. vera* genome with different heterozygosity rates showed it to be about 1% (Fig. 1). We did not observe a fat tail in the K-mer analysis; therefore, the number of repeats in the pistachio genome may be low. The distribution of GC content versus sequencing depth (Fig. 2) may provide information about sequencing bias. The GC content was about 37.1% in pistachio. There is also a region (red region) with an average depth around half that of main average depth, which may be caused by the high rate of heterozygosity.

### Assembly and identification of SSR loci in *P. vera*

Assembly was performed using 26.77-Gb Illumina PE reads. The length of contig N50 was 2327 bp, and the scaffold N50 was 3399-bp long. The total length of scaffolds was 513.5 Mb. The number of scaffolds ≥100 bp was 893,901 and ≥2 kb were 44,900 (Table 1).

**Table 1:** The result of assembly in *P. vera* cv. Siirt using 26.77 Gb Illumina data

	Contigs		Scaffolds	
	Size(bp)	Number	Size(bp)	Number
N90	121	521,093	130	417,692
N80	262	230,751	442	163,608
N70	699	118,780	989	85,774
N60	1325	67,518	1874	47,596
N50^a^	2327	39,274	3399	27,069
Total Size	490,733,307	-	513,504,777	-
Total Number(> = 100 bp)		980,753	-	893,901
Total Number(> = 2 kb)		45,572	-	44,900

^a.^The N50 of contigs or scaffolds was calculated by ordering all sequences, then adding the lengths from the longest to shortest until the added length exceeded 50% of the total length of all sequences.N60, N70, N80 and N90 were similarly defined

**Fig. 3:**
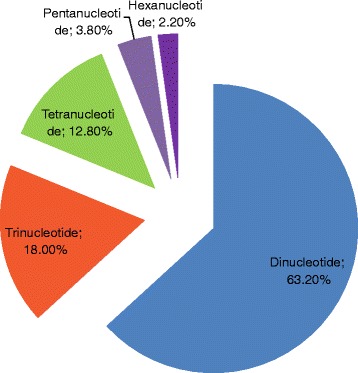
Distribution of 59,280 SSRs in the pistachio genome based on repeat type

**Fig. 4:**
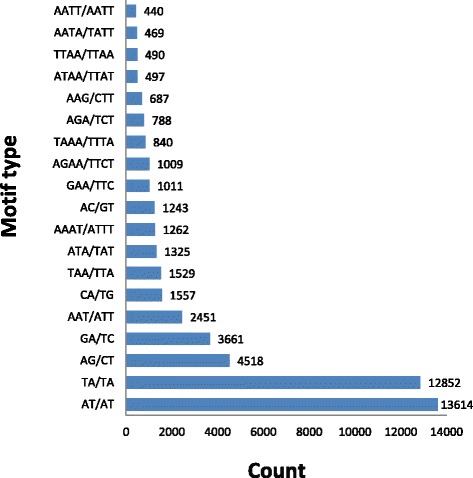
Distribution of SSR motifs in pistachio at 40x coverage sequencing data. The X-axis represents motif types and the Y-axis represents the count of motifs in whole genome of pistachio

A total of 59,280 di-, tri-, tetra-, penta-, and hexanucleotide SSR motifs (6, 5, 4, 4, and 4 repeat numbers, respectively) were detected, and the dinucleotide motifs were the most abundant type of repeats (63.2%) in pistachio (Fig. 3), followed by tri- (18.0%), tetra- (12.8%), penta- (3.8%), and hexanucleotide motifs (2.2%). The most abundant repeats were AT/AT (23.0%) and TA/TA (21.6%), followed by AG/CT (7.6%) and GA/TC (6.2%), AAT/ATT (4.1%), CA/TG (2.6%), and TAA/ATT (2.6%; Fig. 4). The most abundant tetra- and pentanucleotide repeat motif types were AAAT/ATTT (2.1%) and AAAAT/ATTTT (0.44%) respectively. AAAAAT/ATTTTT and GCCCAA/TTGGGC motifs were the most abundant (0.07%) hexanucleotide motifs. The distribution of SSRs in the pistachio genome was calculated as one SSR per 8.67 kb.

### Development, screening, and polymorphism of SSRs

In the initial screen of 950 randomly selected SSR primer pairs in three (*P. vera* cv. Siirt, *P. vera* cv. Bağyolu and one monoecious *P. atlantica*) genotypes, 610 (64.2%) generated amplification products, 197 (20.7%) loci were monomorphic, and the remaining 143 (15.1%) SSR loci failed to generate amplification products. Of the 610 that amplified, 204 polymorphic and easily scorable SSR loci were selected to study genetic diversity in *Pistacia*. Of these, 193 were perfect (94.6%), 8 (3.9%) were compound, and 3 (1.5%) were interrupted repeats. Dinucleotide motifs were the most abundant (63.2%), followed by tri- (18.0%), hexa- (12.8%), tetra- (3.8%), and pentanucleotide motifs (2.2%). The sequences of 204 SSR loci were deposited into NCBI and were given in Additional file 1 (GenBank accession numbers KX223398- KX223601; Additional file 1). Two SSR primer pairs (CUPVSiirt568 and CUPVSiirt689) amplified at two loci, and 206 SSR loci were obtained and used to study genetic diversity in *Pistacia*.

### Diversity measures of novel SSR loci in *Pistacia*

Genetic diversity was studied by analyzing a total of 44 cultivars and genotypes: 24 *P. vera* cultivars and 20 wild *Pistacia* genotypes (four genotypes from each five of wild *Pistacia* species) belonging to *P. atlantica*, *P. integerrima*, *P. chinenesis*, *P. terebinthus*, and *P. lentiscus* genotypes. Allele ranges, number of alleles (Na), effective number of alleles (Ne), polymorphism information content (PIC), expected (He), and observed (Ho) heterozygosities of the 206 SSR loci are presented in Table 2.

**Table 2:** Novel SSR loci with genetic diversity measures in 44 *Pistacia* genotypes: allele ranges, number of alleles (Na), number of effective alleles (Ne), observed heterozygosity (Ho), expected heterozygosity (He), and PIC values of 206 loci

No	Loci	Allele ranges (bp)	Na	Ne	Ho	He	PIC
1	CUPVSiirt15^a^	96–123	10	5.12	0.45	0.80	0.79
2	CUPVSiirt17	103–109	2	1.26	0.00	0.20	0.18
3	CUPVSiirt18	181–185	2	1.09	0.09	0.08	0.08
4	CUPVSiirt22a	134–182	13	8.64	0.46	0.88	0.87
5	CUPVSiirt26^ab^	164–190	9	4.39	0.39	0.77	0.74
6	CUPVSiirt37^ab^	140–167	14	7.43	0.60	0.87	0.85
7	CUPVSiirt45^a^	123–133	2	1.89	0.12	0.47	0.36
8	CUPVSiirt50	155–190	10	4.94	0.33	0.80	0.77
9	CUPVSiirt71^a^	128–168	11	5.75	0.37	0.83	0.81
10	CUPVSiirt76^a^	153–188	8	5.72	0.29	0.83	0.80
11	CUPVSiirt86^a^	111–159	19	8.96	0.82	0.89	0.88
12	CUPVSiirt95^a^	191–235	13	5.74	0.40	0.83	0.81
13	CUPVSiirt115	166–173	6	5.94	0.28	0.83	0.81
14	CUPVSiirt121	106–143	6	2.45	0.10	0.59	0.55
15	CUPVSiirt125^a^	167–224	12	6.73	0.57	0.85	0.83
16	CUPVSiirt129^ab^	127–172	16	6.76	0.59	0.85	0.84
17	CUPVSiirt131^a^	156–175	8	2.96	0.38	0.66	0.62
18	CUPVSiirt140^a^	213–235	7	5.10	0.33	0.80	0.78
19	CUPVSiirt149^a^	98–136	15	5.25	0.43	0.81	0.79
20	CUPVSiirt151	150–172	9	4.17	0.40	0.76	0.73
21	CUPVSiirt158^a^	121–243	9	6.27	0.47	0.84	0.82
22	CUPVSiirt171^a^	138–161	4	2.74	0.25	0.64	0.59
23	CUPVSiirt186^a^	140–182	15	4.42	0.56	0.77	0.76
24	CUPVSiirt213^a^	205–223	7	2.57	0.34	0.61	0.58
25	CUPVSiirt230^a^	177–216	12	4.77	0.30	0.79	0.77
26	CUPVSiirt238	171–195	8	3.80	0.37	0.74	0.70
27	CUPVSiirt242^a^	129–167	13	6.13	0.49	0.84	0.82
28	CUPVSiirt243^a^	137–166	13	4.80	0.41	0.79	0.77
29	CUPVSiirt256^ab^	171–199	7	3.20	0.36	0.69	0.63
30	CUPVSiirt259^a^	186–218	10	5.33	0.58	0.81	0.79
31	CUPVSiirt265^a^	111–200	5	2.97	0.17	0.66	0.62
32	CUPVSiirt271	96–118	10	5.24	0.67	0.81	0.78
33	CUPVSiirt284^ab^	220–256	12	6.08	0.39	0.84	0.82
34	CUPVSiirt294^a^	114–138	10	6.24	0.34	0.84	0.82
35	CUPVSiirt297^a^	136–146	8	5.51	0.30	0.82	0.79
36	CUPVSiirt298	106–198	18	6.86	0.67	0.85	0.84
37	CUPVSiirt308^a^	166–175	8	4.62	0.33	0.78	0.76
38	CUPVSiirt312^a^	154–172	8	2.27	0.29	0.56	0.54
39	CUPVSiirt316	254–280	7	2.87	0.31	0.65	0.62
40	CUPVSiirt320^a^	173–179	2	1.83	0.50	0.45	0.35
41	CUPVSiirt328	181–204	6	2.52	0.40	0.60	0.58
42	CUPVSiirt333^a^	127–159	11	5.92	0.51	0.83	0.81
43	CUPVSiirt340^a^	125–171	12	6.45	0.48	0.85	0.83
44	CUPVSiirt343	121–127	4	1.96	0.33	0.49	0.43
45	CUPVSiirt349^ab^	164–199	13	5.69	0.63	0.82	0.8
46	CUPVSiirt357^a^	181–231	14	9.24	0.60	0.89	0.88
47	CUPVSiirt358	215–269	9	5.80	0.27	0.83	0.81
48	CUPVSiirt368	152–188	7	2.49	0.03	0.60	0.57
49	CUPVSiirt415	140–170	6	3.59	0.28	0.72	0.68
50	CUPVSiirt436^ab^	91–118	13	6.01	0.68	0.83	0.82
51	CUPVSiirt446^a^	221–242	4	1.96	0.36	0.49	0.42
52	CUPVSiirt465^a^	112–178	16	9.93	0.45	0.90	0.89
53	CUPVSiirt472^a^	172–325	16	4.67	0.44	0.79	0.77
54	CUPVSiirt476^ab^	145–171	9	6.01	0.43	0.83	0.82
55	CUPVSiirt479	165–192	5	2.01	0.48	0.50	0.46
56	CUPVSiirt496	158–239	14	3.94	0.26	0.75	0.73
57	CUPVSiirt501	146–171	6	2.26	0.34	0.56	0.52
58	CUPVSiirt505^ab^	146–186	13	4.20	0.43	0.76	0.74
59	CUPVSiirt509^ab^	162–188	12	3.28	0.36	0.69	0.67
60	CUPVSiirt543^a^	111–149	11	6.76	0.63	0.85	0.84
61	CUPVSiirt565^ab^	134–174	16	7.49	0.43	0.87	0.85
62	CUPVSiirt568x	100–117	5	2.46	0.21	0.59	0.52
63	CUPVSiirt568y	117–145	15	8.50	0.48	0.88	0.87
64	CUPVSiirt569^a^	95–109	5	3.99	0.62	0.75	0.71
65	CUPVSiirt598	163–204	14	7.38	0.60	0.86	0.85
66	CUPVSiirt600	225–241	8	5.17	0.54	0.81	0.78
67	CUPVSiirt616	98–204	13	9.88	0.51	0.90	0.89
68	CUPVSiirt617	277–290	4	3.12	0.39	0.68	0.62
69	CUPVSiirt621^a^	93–129	9	3.98	0.33	0.75	0.72
70	CUPVSiirt625^a^	152–191	15	9.05	0.36	0.89	0.88
71	CUPVSiirt649^a^	158–177	4	2.68	0.47	0.63	0.56
72	CUPVSiirt660^ab^	129–146	10	3.89	0.62	0.74	0.71
73	CUPVSiirt661	209–288	7	3.28	0.44	0.70	0.67
74	CUPVSiirt674	235–249	6	2.60	0.32	0.61	0.57
75	CUPVSiirt689x	200–210	5	2.10	0.38	0.52	0.50
76	CUPVSiirt689y	306–314	4	2.18	0.49	0.54	0.45
77	CUPVSiirt690	215–221	2	1.40	0.16	0.28	0.24
78	CUPVSiirt712	180–210	9	3.07	0.46	0.67	0.64
79	CUPVSiirt715	139–176	9	2.64	0.25	0.62	0.59
80	CUPVSiirt719^ab^	190–234	13	6.99	0.71	0.86	0.84
81	CUPVSiirt724	132–138	3	2.88	0.38	0.65	0.58
82	CUPVSiirt742^a^	196–232	14	3.19	0.30	0.69	0.67
83	CUPVSiirt743	159–205	12	6.70	0.39	0.85	0.83
84	CUPVSiirt764^a^	161–206	9	6.14	0.56	0.84	0.82
85	CUPVSiirt768^ab^	206–220	6	3.43	0.60	0.71	0.67
86	CUPVSiirt782^ab^	172–202	10	4.41	0.5	0.77	0.75
87	CUPVSiirt788	205–262	16	6.48	0.43	0.85	0.83
88	CUPVSiirt794	206–244	14	5.90	0.65	0.83	0.81
89	CUPVSiirt796^ab^	92–130	17	8.32	0.66	0.88	0.87
90	CUPVSiirt803	202–266	8	3.49	0.36	0.71	0.68
91	CUPVSiirt818^a^	165–187	10	5.65	0.48	0.82	0.80
92	CUPVSiirt836^a^	144–174	13	2.69	0.23	0.63	0.61
93	CUPVSiirt838^ab^	139–181	14	6.21	0.76	0.84	0.82
94	CUPVSiirt841	146–186	14	5.81	0.59	0.83	0.81
95	CUPVSiirt847	226–256	9	6.13	0.50	0.84	0.82
96	CUPVSiirt855^a^	237–278	9	4.17	0.43	0.76	0.74
97	CUPVSiirt858^a^	167–198	11	4.62	0.63	0.78	0.76
98	CUPVSiirt875	157–190	13	4.23	0.28	0.76	0.74
99	CUPVSiirt876^ab^	180–221	16	8.05	0.77	0.88	0.86
100	CUPVSiirt883	172–219	12	3.69	0.28	0.73	0.71
101	CUPVSiirt889^a^	168–188	8	4.57	0.40	0.78	0.75
102	CUPVSiirt891^a^	130–168	6	3.82	0.28	0.74	0.70
103	CUPVSiirt907	92–98	3	2.86	0.45	0.65	0.58
104	CUPVSiirt924^a^	81–89	4	1.49	0.00	0.33	0.31
105	CUPVSiirt929^a^	81–105	13	5.66	0.40	0.82	0.81
106	CUPVSiirt931^a^	146–340	7	4.59	0.12	0.78	0.75
107	CUPVSiirt932	123–132	7	3.48	0.43	0.71	0.67
108	CUPVSiirt949^a^	160–197	9	4.31	0.34	0.77	0.74
109	CUPVSiirt951	154–184	6	1.78	0.08	0.44	0.42
110	CUPVSiirt956^a^	105–200	18	6.31	0.42	0.84	0.83
111	CUPVSiirt961^a^	177–210	10	5.85	0.33	0.83	0.81
112	CUPVSiirt975^a^	139–154	6	3.44	0.30	0.71	0.66
113	CUPVSiirt986^a^	146–169	10	3.09	0.41	0.68	0.65
114	CUPVSiirt989^a^	140–176	13	4.10	0.32	0.76	0.74
115	CUPVSiirt1003^ab^	71–112	12	7.87	0.49	0.87	0.86
116	CUPVSiirt1008^a^	163–174	6	2.93	0.20	0.66	0.60
117	CUPVSiirt1017^a^	203–245	16	6.14	0.50	0.84	0.82
118	CUPVSiirt1021^a^	123–143	6	3.03	0.57	0.67	0.63
119	CUPVSiirt1041^a^	100–114	7	4.62	0.40	0.78	0.75
120	CUPVSiirt1043^a^	89–157	13	3.43	0.30	0.71	0.69
121	CUPVSiirt1047^ab^	125–157	11	3.74	0.34	0.73	0.72
122	CUPVSiirt1053^a^	145–189	9	4.05	0.40	0.75	0.72
123	CUPVSiirt1055^a^	151–180	7	2.83	0.24	0.65	0.62
124	CUPVSiirt1057^a^	226–258	12	5.05	0.42	0.8	0.78
125	CUPVSiirt1062^a^	138–172	12	3.84	0.56	0.74	0.71
126	CUPVSiirt1071^ab^	123–169	16	7.69	0.63	0.87	0.86
127	CUPVSiirt1092^a^	136–165	11	6.15	0.56	0.84	0.82
128	CUPVSiirt1095	126–290	11	7.78	0.58	0.87	0.86
129	CUPVSiirt1116^a^	151–191	15	5.37	0.57	0.81	0.80
130	CUPVSiirt1117^a^	143–172	12	7.74	0.56	0.87	0.86
131	CUPVSiirt1120^ab^	193–218	11	4.50	0.39	0.78	0.75
132	CUPVSiirt1122	121–215	7	5.54	0.45	0.82	0.80
133	CUPVSiirt1127	118–142	9	4.74	0.50	0.79	0.77
134	CUPVSiirt1140^a^	156–176	8	5.94	0.40	0.83	0.81
135	CUPVSiirt1145^ab^	150–174	12	5.98	0.58	0.83	0.81
136	CUPVSiirt1153^a^	172–201	8	3.96	0.26	0.75	0.72
137	CUPVSiirt1163	140–166	7	3.80	0.43	0.74	0.70
138	CUPVSiirt1171	229–270	14	3.07	0.38	0.67	0.66
139	CUPVSiirt1182^ab^	155–179	8	4.46	0.43	0.78	0.75
140	CUPVSiirt1183	235–250	7	4.48	0.33	0.78	0.75
141	CUPVSiirt1188	116–168	3	2.56	0.31	0.61	0.54
142	CUPVSiirt1189	247–267	5	2.56	0.36	0.61	0.56
143	CUPVSiirt1191	154–190	5	3.19	0.46	0.69	0.65
144	CUPVSiirt1202^ab^	174–212	16	4.18	0.50	0.76	0.74
145	CUPVSiirt1214^a^	157–182	10	4.56	0.15	0.78	0.76
146	CUPVSiirt1224^ab^	257–292	9	6.37	0.53	0.84	0.82
147	CUPVSiirt1238^ab^	229–258	11	3.88	0.45	0.74	0.72
148	CUPVSiirt1243^ab^	134–164	14	6.64	0.20	0.85	0.84
149	CUPVSiirt1250^a^	175–192	10	4.77	0.40	0.79	0.76
150	CUPVSiirt1260^a^	151–184	10	3.21	0.36	0.69	0.66
151	CUPVSiirt1267	124–155	9	4.78	0.49	0.79	0.77
152	CUPVSiirt1271^a^	203–234	10	3.76	0.23	0.73	0.72
153	CUPVSiirt1273^a^	135–160	6	2.53	0.75	0.61	0.55
154	CUPVSiirt1278^a^	176–220	11	2.90	0.21	0.66	0.63
155	CUPVSiirt1322^a^	218–237	9	3.83	0.40	0.74	0.71
156	CUPVSiirt1326^ab^	186–212	17	7.26	0.55	0.86	0.85
157	CUPVSiirt1330	154–201	18	10.58	0.71	0.91	0.90
158	CUPVSiirt1331	97–119	11	4.23	0.34	0.76	0.74
159	CUPVSiirt1345	156–191	7	2.46	0.38	0.59	0.55
160	CUPVSiirt1353	169–197	10	4.70	0.53	0.79	0.76
161	CUPVSiirt1360	127–135	5	3.59	0.34	0.72	0.67
162	CUPVSiirt1372^ab^	112–194	11	4.53	0.20	0.78	0.76
163	CUPVSiirt1378	83–119	11	4.06	0.30	0.75	0.72
164	CUPVSiirt1388^a^	177–201	6	4.67	0.43	0.79	0.75
165	CUPVSiirt1394^a^	228–270	9	5.17	0.28	0.81	0.78
166	CUPVSiirt1399	183–216	6	1.95	0.18	0.49	0.46
167	CUPVSiirt1400^ab^	155–197	17	5.34	0.77	0.81	0.8
168	CUPVSiirt1402^ab^	172–213	9	4.36	0.45	0.77	0.74
169	CUPVSiirt1405^ab^	176–223	18	8.85	0.56	0.89	0.88
170	CUPVSiirt1406	172–208	13	3.81	0.23	0.74	0.72
171	CUPVSiirt1413^a^	166–202	10	3.74	0.46	0.73	0.70
172	CUPVSiirt1417	134–374	12	4.84	0.31	0.79	0.78
173	CUPVSiirt1418^a^	114–159	16	8.24	0.37	0.88	0.87
174	CUPVSiirt1431^a^	198–215	8	3.65	0.29	0.73	0.70
175	CUPVSiirt1438^a^	265–290	9	4.06	0.14	0.75	0.72
176	CUPVSiirt1442^a^	110–138	12	4.12	0.27	0.76	0.74
177	CUPVSiirt1457^a^	149–199	12	7.84	0.32	0.87	0.86
178	CUPVSiirt1477^ab^	109–126	12	6.27	0.57	0.84	0.83
179	CUPVSiirt1478	84–119	11	4.77	0.46	0.79	0.76
180	CUPVSiirt1517^a^	213–225	10	5.13	0.21	0.8	0.79
181	CUPVSiirt1547^a^	120–126	2	1.38	0.18	0.28	0.24
182	CUPVSiirt1564	188–222	12	5.94	0.34	0.83	0.81
183	CUPVSiirt1567^ab^	181–215	15	5.69	0.52	0.82	0.81
184	CUPVSiirt1599	292–345	11	6.53	0.39	0.85	0.83
185	CUPVSiirt1611^ab^	193–215	8	2.68	0.16	0.63	0.6
186	CUPVSiirt1626^a^	114–153	12	7.14	0.27	0.86	0.85
187	CUPVSiirt1628	125–155	7	2.98	0.40	0.66	0.61
188	CUPVSiirt1639^a^	167–194	7	4.60	0.39	0.78	0.75
189	CUPVSiirt1640^a^	134–180	10	3.45	0.17	0.71	0.67
190	CUPVSiirt1652^ab^	159–193	13	5.30	0.61	0.81	0.79
191	CUPVSiirt1655^ab^	150–213	14	5.77	0.53	0.83	0.81
192	CUPVSiirt1658^a^	130–209	13	3.89	0.44	0.74	0.72
193	CUPVSiirt1667^ab^	149–186	12	4.58	0.42	0.78	0.76
194	CUPVSiirt1688	160–184	11	5.74	0.63	0.83	0.80
195	CUPVSiirt1705	231–251	3	1.35	0.06	0.26	0.24
196	CUPVSiirt1714	196–208	5	2.13	0.08	0.53	0.50
197	CUPVSiirt1734	168–185	5	3.24	0.50	0.69	0.65
198	CUPVSiirt1740^a^	157–192	7	2.88	0.41	0.65	0.61
199	CUPVSiirt1742^a^	149–213	15	7.32	0.25	0.86	0.85
200	CUPVSiirt1749^ab^	133–164	9	4.44	0.52	0.77	0.74
201	CUPVSiirt1759^ab^	137–159	11	5.79	0.62	0.83	0.81
202	CUPVSiirt1764^a^	154–178	13	3.59	0.43	0.72	0.71
203	CUPVSiirt1768^a^	108–128	8	3.63	0.45	0.72	0.69
204	CUPVSiirt1784^a^	177–211	9	5.46	0.43	0.82	0.79
205	CUPVSiirt1788^a^	172–189	4	2.44	0.14	0.59	0.51
206	CUPVSiirt1797^a^	143–177	13	4.98	0.45	0.8	0.78
	Total		2036	-	-	-	-
	Mean		9.88	4.67	0.41	0.74	0.71

^a^had amplifications in six *Pistacia* species

^b^were polymorphic in six *Pistacia* species

A total of 2036 alleles were produced by 206 SSR loci in 44 *Pistacia* cultivars and genotypes, ranging from 2 to 19 per locus. The highest number of alleles was obtained from the CUPVSiirt86 locus. The CUPVSiirt298, CUPVSiirt956, CUPVSiirt1330, and CUPVSiirt1405 loci also produced a high number of alleles (Na = 18). The effective number of alleles ranged from 1.09 (CUPVSiirt18) to 10.58 (CUPVSiirt1330) with an average of Ne = 4.67. The CUPVSiirt465 (Ne = 9.93), CUPVSiirt616 (Ne = 9.88), CUPVSiirt357 (Ne = 9.24), and CUPVSiirt625 (Ne = 9.05) loci also had high effective numbers of alleles. The observed heterozygosity (Ho) ranged from 0.0 to 0.82 with an average of Ho = 0.41. The CUPVSiirt86 locus was the most heterozygous, whereas the CUPVSiirt17 and CUPVSiirt924 loci were homozygous. The average He value was 0.74, which ranged between 0.08 (CUPVSiirt18) and 0.91 (CUPVSiirt1330). The PIC values ranged from 0.08 to 0.90, with an average of 0.71 (Table 2).

### Diversity of the SSRs in each of six *Pistacia* species

In *P. vera*, all 206 SSR loci generated amplification products, and a total of 897 alleles were produced with an average of 4.5 alleles per locus. Two-hundred (97.1%) SSR loci were polymorphic in 24 pistachio cultivars. The highest number of allele (Na = 11) was obtained from the CUPVSiirt1330 locus. The effective number of alleles ranged from 1.04 to 7.60 (CUPVSiirt616). The average observed heterozygosity (Ho) was 0.46, and the CUPVSiirt86 and CUPVSiirt1273 loci were the most heterozygous. The highest expected heterozygosity (0.87) and PIC (0.85) values were produced from the CUPVSiirt616 locus. The average He and PIC values in *P. vera* were calculated as 0.55 and 0.50, respectively (Additional file 2).

In *P. atlantica*, 200 SSR loci generated amplification products with a high rate of transferability (97.1%). Thirty-nine (19.5%) of the amplified SSR loci were monomorphic and the rest were polymorphic (80.5%). A total of 527 alleles were produced by 161 polymorphic SSR loci, with an average of 3.3 alleles per locus. The average observed heterozygosity (Ho) was 0.48. The highest number of alleles (Na = 7), effective number of alleles (Ne = 6.4), expected heterozygosity (0.84), and PIC (0.82) values were produced from the CUPVSiirt349, CUPVSiirt841, and CUPVSiirt1400 loci. The average He and PIC values in *P. atlantica* were 0.56 and 0.49, respectively (Additional file 3).

In *P. integerrima*, the transferability of SSR loci was also high, with a rate of 93.7%. Of the amplified SSR loci, 157 (81.3%) were polymorphic in *P. integerrima*. A total of 416 alleles were produced by 157 SSR loci with an average of 2.70 alleles per locus, and the highest number (Na = 5) of alleles was obtained from the CUPVSiirt131, CUPVSiirt742, CUPVSiirt838, and CUPVSiirt1330 loci. The highest effective number of alleles (4.57) was calculated at the CUPVSiirt742 locus. The average observed (Ho) and expected (He) heterozygosities were 0.50 and 0.52, respectively. The highest values for expected heterozygosity (0.78) and PIC (0.75) were produced from the CUPVSiirt742 locus. The average PIC value in *P. integerrima* was 0.44 (Additional file 4).

In *P. terebinthus*, 183 SSR loci (88.8%) generated amplification products and 142 (77.6%) were polymorphic. A total of 416 alleles were produced by 142 polymorphic SSR loci*,* ranging from 1 to 7 with an average of 3.4 alleles per locus. The effective number of alleles ranged from 1.28 to 6.40. The observed heterozygosity (Ho) ranged from 0.0 to 1.0, with an average of 0.47. The highest number of alleles (Na = 7), effective number of alleles (Ne = 6.4), expected heterozygosity (0.84), and PIC (0.82) were obtained from the CUPVSiirt1017, CUPVSiirt1326, CUPVSiirt1405, and CUPVSiirt1406 loci. The average He and PIC values for *P. terebinthus* were 0.56 and 0.50, respectively (Additional file 5).

In *P. chinensis*, 177 (85.9%) loci amplified in SSR-PCR analysis, and 119 loci (67.2%) were polymorphic. A total of 365 alleles were amplified from 119 polymorphic SSR loci with an average of 3.1 alleles per locus*.* The average observed heterozygosity (Ho) was 0.48. The highest values for He and PIC were 0.84 and 0.82, respectively. The CUPVSiirt836 locus amplified the highest number of alleles and had the highest level of polymorphism. The average values for He and PIC in *P. chinensis* were 0.54 and 0.48, respectively (Additional file 6).

In *P. lentiscus*, 151 (73.3%) SSR loci amplified, with the lowest transferability among the five wild *Pistacia* species studied. Of the amplified SSR loci, 83 (55.0%) were polymorphic. A total of 217 alleles were obtained by 83 polymorphic SSR loci in *P. lentiscus,* ranging from 1 to 6, with an average of 2.6 alleles per locus. The effective number of alleles ranged from 1.28 to 4.57. The observed heterozygosity (Ho) ranged from 0 to 1 with an average of 0.50. The average values for He and PIC in *P. lentiscus* were 0.49 and 0.41, respectively. The highest values for Na, Ne, He, and PIC values were obtained from the CUPVSiirt1797 locus (Additional file 7).

Of the 206 SSR loci analyzed in this study, 136 generated amplifications and 41 were polymorphic in all six *Pistacia* species. Mean values for genetic parameters in each of the *Pistacia* species are shown in Table 3. The highest transferability was obtained in *P. atlantica* and *P. integerrima,* while *P. lentiscus* had the lowest transferability. In wild *Pistacia* species, the highest average number of alleles was obtained in *P. atlantica* and *P. terebinthus*, while *P. lentiscus* had the lowest number.

**Table 3:** Mean of population genetic parameters of polymorphic SSR loci in each of *Pistacia* species

Species	Transferability (%)	Total polymorphic number of alleles	Na	Ne	Ho	He	PIC
ALL	206 (100%)	2036	9.88	4.67	0.41	0.74	0.71
*P. vera*	206 (100%)	897	4.48	2.57	0.46	0.55	0.50
*P. atlantica*	200 (97.1%)	527	3.27	2.59	0.48	0.56	0.49
*P. integerrima*	193 (93.7%)	416	2.64	2.21	0.50	0.52	0.44
*P. chinensis*	177 (86.0%)	365	3.06	2.51	0.48	0.54	0.48
*P. terebinthus*	183 (88.9%)	485	3.41	2.80	0.47	0.56	0.50
*P. lentiscus*	151 (73.3%)	217	2.61	2.13	0.50	0.49	0.41

### Cluster analysis and genetic structure

**Fig. 5:**
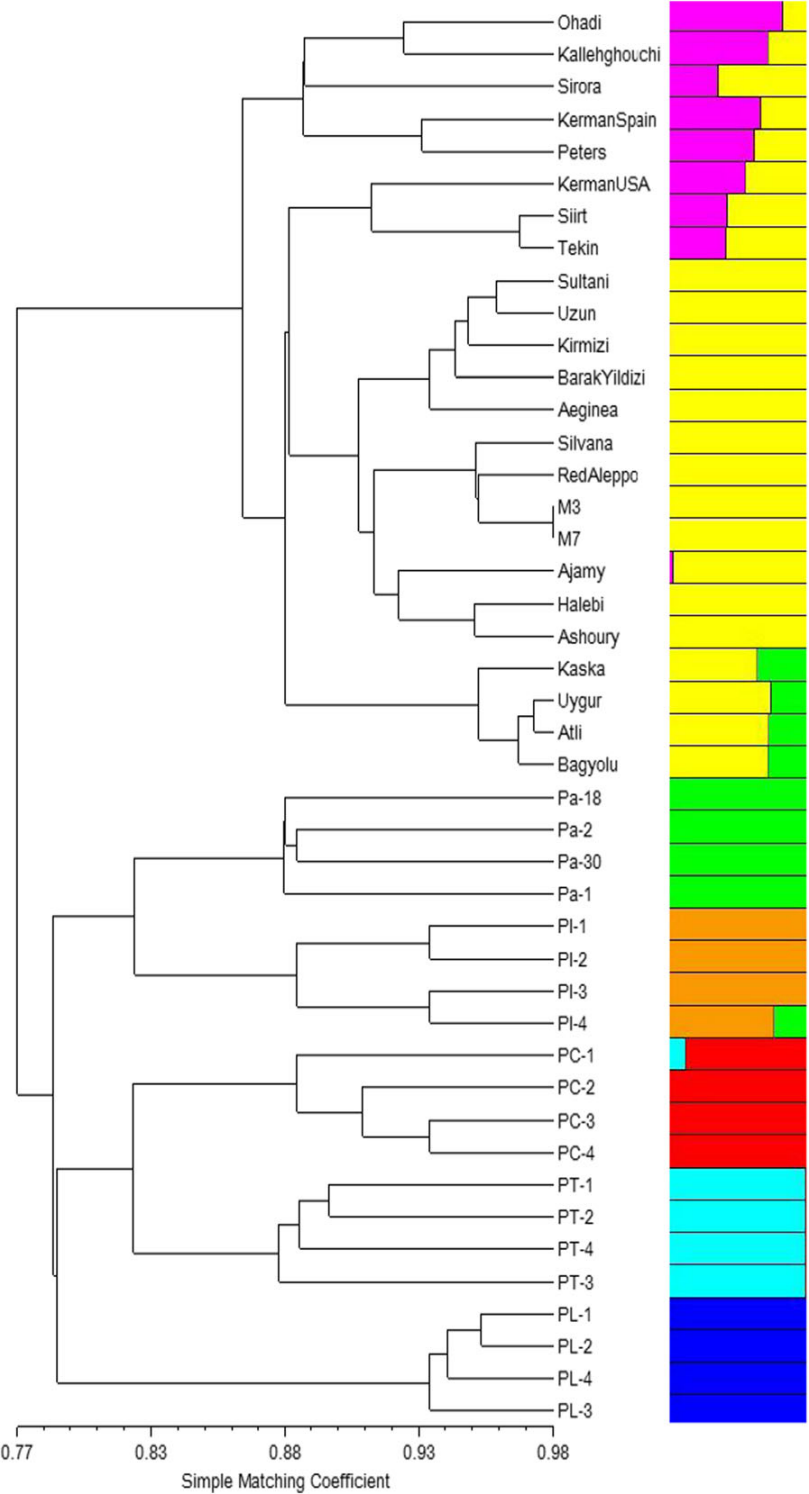
UPGMA dendrogram of 24 *P. vera* cultivars and 20 genotypes belongs to *P. atlantica*, *P. integerrima*, *P. terebinthus*, *P. chinensis* and *P. lentiscus.* The colors refers to population structure when K = 6, which all six *Pistacia* species were clearly separated

Cluster analysis was performed for 24 *P. vera* cultivars and 20 wild *Pistacia* genotypes using 136 SSR loci that amplified in all tested *Pistacia* species. UPGMA analysis showed that all *Pistacia* species and genotypes were clearly separated from each other. Two main clusters were observed: the first cluster contained all individuals from *P. vera*, whereas the second cluster included wild *Pistacia* species: *P. atlantica*, *P. integerrima*, *P. chinensis*, *P. terebinthus,* and *P. lentiscus* (Fig. 5). *P. atlantica* was the closest species to *P. vera*, while *P. lentiscus* was the most distant.

**Fig. 6:**
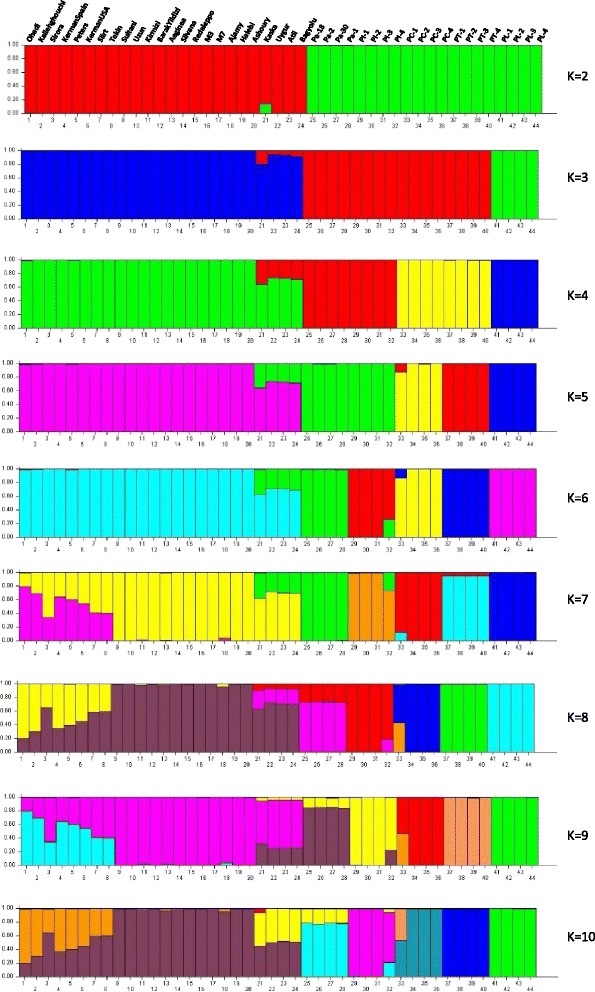
Population structure of 24 *P. vera* cultivars and twenty wild genotypes belong to *P. atlantica*, *P. integerrima*, *P. terebinthus*, *P. chinensis* and *P. lentiscus*. K = 2 to K = 10 represent the sub-populations

**Fig. 7:**
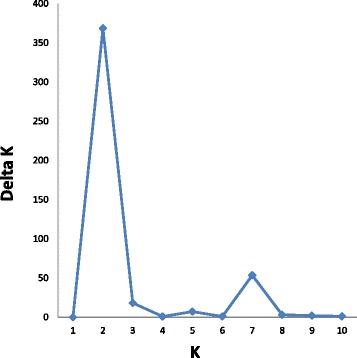
Values of DK. The modal value of this distribution is the true K(*) or the uppermost level of structure, here two (K = 2) clusters with 7 sub-clusters (K = 7)

The genetic structure of the *Pistacia* genotypes used in this study is shown in Fig. 6. A model-based clustering method was performed for all 44 genotypes using 136 SSR loci. The most probable number of clusters was identified by calculating the Delta K (ΔK), which is based on the rate of change in the log probability of data between successive K values (K = 1 to K = 10). The peak of the ΔK graph corresponds to the most probable number of populations in the data set. The highest number of delta K (ΔK) was found at K  =  2 (Fig. 7), where all 44 genotypes were divided into two main groups similar to the UPGMA dendrogram (Fig. 5). As the value for K increased to 3, the genotypes in group 2 were divided into two sub-groups: the first subgroup contained *P. lentiscus* and *P. terebinthus* and the second subgroup contained the other wild *Pistacia* species. When K = 4, the wild *Pistacia* genotypes were divided into three subgroups: the first group included *P. lentiscus*, the second group contained *P. terebinthus,* and *P. chinensis*, and the third group contained *P. atlantica* and *P. integerrima*. When K = 5, all *Pistacia* species were separated from each other, with the exception of *P. terebinthus* and *P. chinensis.* When K = 6, all six *Pistacia* species were clearly separated. When K = 7, the *Pistacia* species were again divided into six groups, and *P. vera* cultivars were grouped based on their origins, which was supported by another high ΔK was found at K = 7 (Figs. 5, 6 and 7). The cultivars originated from Iran were in one cluster, and the other cultivars were in the other cluster.

## Discussion

### Genome size, heterozygosity, and GC content

The development of NGS technology has provided researchers with an affordable way of addressing a wide range of questions, especially in non-model species such as pistachio. In addition, the K-mer method has been successfully applied for the estimation of genome size using NGS reads without prior knowledge of the genome size [32]. This approach has been used to analyze a number of plant genomes such as in switchgrass (*Panicum virgatum* L.) [33], Chinese bayberry (*Myrica rubra* Sieb. et Zucc.) [26], Chinese jujube (*Ziziphus jujuba* Mill) [34], and *Rosa roxburghii* Tratt [32]. Here, for the first time, we report a genome survey of *P. vera* using whole genome shotgun sequencing. The 17-nucleotide depth distribution suggested that the genome size of *P. vera* is about 600 Mb, which is close to the size (660 Mb) previously estimated for *P. terebinthus* using flow cytometry [28]. The estimated genome size of pistachio was found to be smaller than that of apple [35], and larger than that of peach [36], sweet orange [37], and poplar [38]. The small size of the pistachio genome may encourage scientists to perform whole genome sequencing in this species. The K-mer analysis also suggested a high level of heterozygosity for *P. vera*, which is probably due to the dioecious mating system in this genus. Information about the genome structure of pistachio from this study may be useful for whole genome sequencing in these plants.

The average GC content of the *P. vera* genome was higher than that of wild sweet potato (36.0%) [39], but lower than that of switchgrass (45.5%) [33] and Chinese jujube (48%) [34]. Different GC contents may result in sequencing bias on the Illumina sequencing platform, and can, therefore, seriously affect genome assembly [40, 41]. GC content was one of three factors found to contribute to sequencing bias on the Illumina sequencing platform [42]. High and low GC contents result in reduced coverage in sequencing regions [41].

### SSR polymorphisms in *Pistacia*

From the 59,280 SSRs detected in the genome survey of pistachio in this study, primer design was performed for 950 loci. Initial screening of these loci for polymorphisms and ease of scoring revealed that 206 SSR loci were polymorphic and had good amplifications in genetic diversity analyses of 44 *Pistacia* genotypes. In *P. vera*, 200 SSR loci were polymorphic and can be used in further studies investigating genetic linkage mapping, germplasm characterization, fingerprinting, and genetic diversity. Several reports have been published on SSR development in *P. vera* [11, 19–21]. Those authors reported a total of 137 polymorphic SSR loci in *P. vera*. Therefore, the number of polymorphic SSR loci developed in the present study was higher than that previously reported for *P. vera*.

Wild *Pistacia* species have been used commonly for rootstock seed sources and forest trees. We also tested 206 SSR loci in five wild *Pistacia* species for their possible use in genetic diversity studies. We report polymorphic SSR loci for each species: 161 for *P. atlantica*, 157 for *P. integerrima*, 142 for *P. terebinthus*, 119 for *P. chinensis*, and 83 polymorphic for *P. lentiscus*. Albaladejo et al. [22] only developed eight polymorphic SSR loci for *P. lentiscus*. Zaloglu et al. [20] and Topcu et al. [11] developed SSRs from *P. vera* and tested them for PCR amplification in wild *Pistacia* species. The authors did not analyze the SSR loci for polymorphism. Therefore, this study describes an important number of novel polymorphic SSRs for each of five wild *Pistacia* species.

The transferability rates of SSRs in this study were high; this is consistent with findings from previous studies performed by Zaloglu et al. [20] and Topcu et al. [11]. Taken together, these data demonstrate that SSRs are very powerful tools for use in synteny analysis in *Pistacia*. The lowest transferability rate was obtained for *P. lentiscus*, which is one of the most distant species to *P. vera* in the genus [1, 2]. The average number of alleles (Na = 4.5) in *P. vera* was higher in this study, while PIC, observed (Ho), and expected (He) heterozygosities were similar to the values obtained in previous SSR development studies [11, 19–21].

The first genetic linkage map in pistachio was constructed by Türkeli and Kafkas [13] using an F1 interspecific population between *P. vera* (cv Siirt) and monoecious *P. atlantica* (Pa-18). The same cultivar and the monoecious genotype were also plant materials of this study. All the 206 SSR loci in this study were polymorphic between Siirt cultivar and monoecious Pa-18 genotype. Türkeli and Kafkas [13] constructed 17 and 19 linkage groups in Siirt and Pa-18, respectively, that were higher than haploid chromosome number of pistachio, *n* = 15 [43]. Therefore, the polymorphic SSR loci developed in this study may facilitate to construct a reference SSR-based linkage map of pistachio.

### Cluster analysis and genetic structure in *Pistacia*

All the genotypes and all six *Pistacia* species separated and consistently grouped well in both cluster and structure analysis. The dendrogram and structure analysis at K = 2 divided *Pistacia* species into two main clusters: the first cluster included all *P. vera* cultivars, while the second cluster contained the wild genotypes belonging to five *Pistacia* species. The second cluster divided into two subclusters: the first subcluster contained *P. atlantica* and *P. integerrima* species, while the second subcluster contained *P. chinensis*, *P. terebinthus,* and *P. lentiscus. P. atlantica* was the closest species to *P. vera*, whereas *P. lentiscus* was the most distant species. Similar results were obtained from other studies undertaking phylogenetic analyses in the genus *Pistacia* [1, 2, 9]. Kafkas and Perl-Treves [44] divided the genus into two main groups: the first group included species with large, single-trunked trees, whereas the second group included species that mostly grow as shrubs or small trees. *P. terebinthus* and *P. lentiscus* were in the same group. These species were also clustered in the same group and were found to be the closest species in this study. A similar result was reported by Kafkas [1] using AFLP markers.

The most comprehensive study on the relationships between *P. vera* cultivars was performed by Kafkas et al. [14]. Those authors grouped 69 *P. vera* cultivars into two main groups: the first contained the cultivars originated from Iran and the second included the cultivars from Mediterranean countries such as Turkey, Syria, and Greece. We also observed similar groupings and genetic relationships among 24 pistachio cultivars in this study. Structure analysis at K = 7 also supported the findings of the previous study performed by Kafkas et al. [14], and was confirmed by the UPGMA clustering analysis in this study. Iranian cultivars such as Ohadi and Kallehghouchi were clustered in the Iranian group while the other cultivars were clustered in the Mediterranean group. The Australian cultivar, Sirora was in the Iranian group, and this was supported by structure analysis at K = 7. There were two Kerman clones in the germplasm of the Pistachio Research Institute, which were introduced from Spain and USA. The clone introduced from Spain was clustered in the Iranian group and the other had a close relationship with the Siirt cultivar from Turkey. Further study is necessary to elucidate the real Kerman. The male cultivars Atli, Uygur, Bagyolu, and Kaska were in the separate group within *P. vera* in the UPGMA dendrogram. The structure analysis demonstrated that these cultivars have a wild origin as they share an important number of alleles with the wild *Pistacia* species.

## Conclusions

We had approximately 40× Illumina data coverage for the genome survey in *P. vera* to gain knowledge on the structure of the pistachio genome. The assembled data were also used to search for SSRs in the pistachio genome, to develop novel SSR markers, and to study genetic diversity in six *Pistacia* species. K-mer analysis indicated that the pistachio genome is highly heterozygous and is about 600 Mb in size. The level of repeats in the pistachio genome was not high and the GC content was about 37.1%. The SSR search in the assembled genome revealed 59,280 SSRs with a frequency of 8.67 kb. A total of 206 polymorphic SSR loci were developed from 950 SSR loci: 136 had amplifications and 41 were polymorphic in all six *Pistacia* species. In conclusion, in this study, we present the first data on the structure of the pistachio genome, which may help to design whole genome sequencing studies in pistachio. Furthermore, we also report novel polymorphic SSR markers for six *Pistacia* species, which will enable further genetic mapping, genetic diversity, and germplasm characterization studies to be performed in the genus.

## Methods

### Plant materials and DNA extraction

For the genome survey study, DNA from the *P. vera* cv. Siirt cultivar was sequenced using an Illumina (Hi-Seq 2000) next generation sequencing platform. Twenty-four *P. vera* cultivars (Kerman-USA, Kerman-Spain, Peters, Kallehghouchi, Sultani, Kaska, Uzun, Kirmizi, Barak Yildizi, Uygur, Atli, Tekin, Ajamy, Aeginea, Halebi, Ashoury, M3, M7, Silvana, Sirora, Red Aleppo, Siirt, Ohadi, and Bagyolu) were used to test SSR markers for polymorphism and to assess genetic diversity. The origins of the cultivars were as previously described by Kafkas et al. [14]. fresh leaves of 20 wild *Pistacia* genotypes (four genotypes from each five wild *Pistacia* species) belonging to *P. atlantica*, *P. integerrima*, *P. terebinthus*, *P. chinensis,* and *P. lentiscus* were collected to test transferability of SSR markers across *Pistacia* species and to analyze genetic diversity within each species.

About 4–5 g fresh leaves were sampled from germplasm collections of Çukurova University in Adana and Pistachio Research Institute in Gaziantep. Genomic DNA was extracted using the CTAB protocol [45] with minor modifications as described by Kafkas et al. [46]. DNA concentrations were measured using a Qubit Fluorimeter (Invitrogen) or were estimated by comparing the band intensity with λ DNA of known concentrations following 0.8% agarose gel electrophoresis and ethidium bromide staining. DNA samples were subsequently diluted to a concentration of 10 ng/μL for SSR-polymerase chain reaction (PCR).

### Genome survey and microsatellite identification

For the genome survey study, 26.77 Gb clean data were generated after removing low quality reads from two different libraries: 18.72 Gb data was from a 250-bp library with 150-bp pair-end (PE) reads and 8.05 Gb data was from a 500-bp library with 90-bp PE reads. The library constructions and sequencing were performed at the Beijing Genomic Institute, China. All data were used to perform K-mer analysis. Based on the results of the K-mer analysis, information on peak depth and the number of 17-mers was obtained and used to estimate the size of the genome, repetitive sequences, and heterozygosity. Its relationship was expressed by using the following algorithm: Genome size = K-mer num/Peak depth, where the K-mer_num is the total number of K-mer, and Peak_depth is the expected value of K-mer depth. Assembly was performed using SOAPdenovo v2.01 software [47] and the GC depth distribution was determined by SOAPaligner v2.21 [48].

SSR loci were searched using SSRIT [49] software. The search parameters were set for the detection of di-, tri-, tetra-, penta-, and hexanucleotide SSR motifs with a minimum of 6, 5, 4, 4, and 4 repeats, respectively. The SSR loci were subjected to primer design using Primer 3 web based software [50] with the standard parameters.

### Primer selection and PCR conditions

A total of 950 randomly selected primer pairs were synthesized and used for SSR development. PCR and capillary electrophoresis were performed to initially screen SSR primer pairs for polymorphism using two *P. vera* cultivars (Siirt and Bağyolu) and one monoecious *P. atlantica* genotype (Pa-18), which are parents in our monoecious cultivar breeding program. Then, 204 SSR primer pairs were selected for further studies based on their polymorphism and ease of scoring.

SSR-PCR was carried out using a three primer strategy according to the method described by Schuelke [51] with some modifications. PCR was performed in a total volume of 12.5 μL containing 20 ng DNA, 75 mM Tris–HCl (pH 8.8), 20 mM (NH_4_)_2_SO_4_, 2.0 mM MgCl_2_, 0.01% Tween 20, 200 μM each dNTP, 10 nM M13 tailed forward primer at the 5 end, 200 nM reverse primer, 200 nM universal M13 tail primer (5 TGTAAAACGACGGCCAGT-3) labeled with one of FAM, VIC, NED, or PET dyes, and 0.6 U hotstart *Taq* DNA polymerase.

Amplification was performed in two steps as follows: initial denaturation at 94 °C for 3 min, followed by 10 cycles at 94 °C for 30 s, 58 °C for 45 s, and 72 °C for 60 s. The second step included 30 cycles at 94 °C for 30 s, 58 °C for 45 s and 72 °C for 60 s, and a final extension at 72 °C for 10 min. When the PCR was completed, the reactions were subjected to denaturation for capillary electrophoresis in an ABI 3130xl genetic analyzer [Applied Biosystems Inc., Foster City, Calif. (ABI)] using a 36-cm capillary array with POP7 as the matrix (ABI). Samples were fully denatured by mixing 0.5 μL of the amplified product with 0.2 μL of the size standard and 9.8 μL formamide. The fragments were resolved using ABI data collection software 3.0, and SSR fragment analysis was performed with GeneScan Analysis Software 4.0 (ABI).

SSR markers were prefixed with CUPVSiirt; CU denotes Cukurova University and PVSiirt denotes the *Pistacia vera* cv. Siirt, from which the SSRs were isolated. Following digits were obtained from the SSR number, x and y were used to identify different SSR loci produced by the same primer pair.

### Data analysis

The 204 SSR primer pairs selected in the initial screening were used to evaluate the genetic diversity of 24 *P. vera* cultivars. The SSR loci were also tested to determine their genetic diversity and transferability to *P. atlantica*, *P. terebinthus*, *P. integerrima*, *P. chinensis,* and *P. lentiscus* species. Transferability of the SSR markers was calculated for each *Pistacia* species by comparing the number of amplified loci with the total number of loci analyzed. Number of alleles (Na), number of effective alleles (Ne), observed (Ho), and expected (He) heterozygosity were calculated using GenAlEx version 6.5 [52]. The polymorphism information contents (PIC) of each locus was calculated using PowerMarker software version 3.25 [53]. A dendrogram was obtained using NTSYSpc v2.21c [54] software by unweighted pair-group method with arithmetic averages (UPGMA).

STRUCTURE 2.3.4 software [55] was also used to determine the number of populations and for construction of the population structure. The burn-in period and Markov chain Monte Carlo (MCMC) were set at 50,000 and 500,000, respectively. The average value of ln likelihood when K changed from 1 to 10 was calculated according to their genetic similarity, and each run was replicated five times to ensure consistency of results.

## Additional files

Additional file 1: The SSR sequences, accession numbers, forward and reverse primers, repeat motif, repeat type, and product sizes of 204 novel SSR loci developed from *Pistacia vera* L. ‘Siirt’. (XLSX 37 kb)
Additional file 2: Genetic diversity measures in *P. vera*: allele ranges, number of alleles (Na), number of effective alleles (Ne), observed heterozygosity (Ho), expected heterozygosity (He), and polymorphic information content (PIC) of 200 polymorphic SSR loci. (DOCX 40 kb)
Additional file 3: Genetic diversity measures in *P. atlantica*: allele ranges, number of alleles (Na), number of effective alleles (Ne), observed heterozygosity (Ho), expected heterozygosity (He), and PIC values of 161 polymorphic SSR loci. (DOCX 37 kb)
Additional file 4: Genetic diversity measures in *P. integerrima*: allele ranges, number of alleles (Na), number of effective alleles (Ne), observed heterozygosity (Ho), expected heterozygosity (He), and PIC values of 157 polymorphic SSR loci. (DOCX 37 kb)
Additional file 5: Genetic diversity measures in *P. chinensis*: allele ranges, number of alleles (Na), number of effective alleles (Ne), observed heterozygosity (Ho), expected heterozygosity (He), and PIC values of 142 polymorphic SSR loci. (DOCX 37 kb)
Additional file 6: Genetic diversity measures in *P. terebinthus*: allele ranges, number of alleles (Na), number of effective alleles (Ne), observed heterozygosity (Ho), expected heterozygosity (He), and PIC values of 119 polymorphic SSR loci. (DOCX 38 kb)
Additional file 7: Genetic diversity measures in *P. lentiscus*: allele ranges, number of alleles (Na), number of effective alleles (Ne), observed heterozygosity (Ho), expected heterozygosity (He), and PIC values of 83 polymorphic SSR loci. (DOCX 34 kb)

## Abbreviations

AFLP: Amplified fragment length polymorphism
CTAB: Cetyltrimethylammonium bromide
He: Expected heterozygosity
Ho: Observed heterozygosity
ISSR: Inter-simple sequence repeats
Na: Number of alleles
Ne: Number of effective alleles
PIC: Polymorphism information content
RAPD: Randomly amplified polymorphic DNA
SAMPL: Selectively amplified microsatellite polymorphic loci
SNP: Single nucleotide polymorphism
SRAP: Sequence-related amplified polymorphism
SSR: Simple sequence repeat
UPGMA: unweighted pair-group method with arithmetic averages
