# Supplementary material for: Genome survey of pistachio (Pistacia vera L.) by next generation sequencing: Development of novel SSR markers and genetic diversity in Pistacia species
Source: BMC Genomics. 2016 Dec 7;17:998. doi: 10.1186/s12864-016-3359-x (PMC5142174; doi:10.1186/s12864-016-3359-x)
Supplement: Additional file 2: — Genetic diversity measures in P. vera: allele ranges, number of alleles (Na), number of effective alleles (Ne), observed heterozygosity (Ho), expected heterozygosity (He), and polymorphic information content (PIC) of 200 polymorphic SSR loci. (DOCX 40 kb) [file 12864_2016_3359_MOESM2_ESM.docx]

**Additional file 2. Number of alleles (Na), Number of effective alleles (Ne), observed heterozygosity (Ho), expected heterozygosity (He), PIC values and allele range of 200 polymorphic SSR loci developed from *Pistacia vera.***

| **No** | **Loci** | **Na** | **Ne** | **Ho** | **He** | **PIC** | **Alele range**  **(bp)** |
| --- | --- | --- | --- | --- | --- | --- | --- |
| 1 | CUPVSiirt15 | 4 | 2.00 | 0.63 | 0.50 | 0.45 | 106-115 |
| 2 | CUPVSiirt18 | 2 | 1.09 | 0.09 | 0.08 | 0.08 | 181-185 |
| 3 | CUPVSiirt22 | 8 | 3.54 | 0.53 | 0.72 | 0.69 | 162-180 |
| 4 | CUPVSiirt26 | 5 | 3.01 | 0.30 | 0.67 | 0.60 | 179-190 |
| 5 | CUPVSiirt37 | 6 | 2.98 | 0.63 | 0.66 | 0.61 | 143-157 |
| 6 | CUPVSiirt45 | 2 | 1.63 | 0.24 | 0.39 | 0.31 | 123-133 |
| 7 | CUPVSiirt50 | 6 | 3.29 | 0.41 | 0.70 | 0.66 | 162-190 |
| 8 | CUPVSiirt71 | 6 | 2.41 | 0.68 | 0.59 | 0.56 | 156-168 |
| 9 | CUPVSiirt76 | 4 | 2.78 | 0.27 | 0.64 | 0.59 | 153-188 |
| 10 | CUPVSiirt86 | 9 | 5.10 | 1.00 | 0.80 | 0.78 | 142-159 |
| 11 | CUPVSiirt95 | 6 | 3.35 | 0.46 | 0.70 | 0.67 | 193-226 |
| 12 | CUPVSiirt115 | 5 | 4.66 | 0.25 | 0.79 | 0.75 | 169-173 |
| 13 | CUPVSiirt121 | 2 | 1.04 | 0.04 | 0.04 | 0.04 | 106-143 |
| 14 | CUPVSiirt125 | 5 | 3.65 | 0.75 | 0.73 | 0.68 | 167-187 |
| 15 | CUPVSiirt129 | 4 | 2.84 | 0.46 | 0.65 | 0.58 | 141-163 |
| 16 | CUPVSiirt131 | 2 | 1.44 | 0.38 | 0.30 | 0.26 | 163-169 |
| 17 | CUPVSiirt140 | 4 | 2.92 | 0.50 | 0.66 | 0.59 | 215-235 |
| 18 | CUPVSiirt149 | 4 | 1.98 | 0.38 | 0.50 | 0.42 | 105-116 |
| 19 | CUPVSiirt151 | 3 | 2.17 | 0.42 | 0.54 | 0.47 | 154-166 |
| 20 | CUPVSiirt158 | 6 | 3.49 | 0.79 | 0.71 | 0.66 | 227-243 |
| 21 | CUPVSiirt171 | 2 | 1.35 | 0.22 | 0.26 | 0.22 | 149-161 |
| 22 | CUPVSiirt186 | 7 | 2.57 | 0.70 | 0.61 | 0.58 | 155-182 |
| 23 | CUPVSiirt213 | 2 | 1.38 | 0.33 | 0.28 | 0.24 | 215-219 |
| 24 | CUPVSiirt230 | 4 | 2.23 | 0.25 | 0.55 | 0.51 | 185-195 |
| 25 | CUPVSiirt238 | 6 | 2.69 | 0.46 | 0.63 | 0.59 | 173-195 |
| 26 | CUPVSiirt242 | 8 | 3.21 | 0.53 | 0.69 | 0.66 | 138-167 |
| 27 | CUPVSiirt243 | 3 | 1.88 | 0.38 | 0.47 | 0.41 | 142-146 |
| 28 | CUPVSiirt256 | 5 | 2.76 | 0.46 | 0.64 | 0.58 | 171-187 |
| 29 | CUPVSiirt259 | 5 | 2.22 | 0.71 | 0.55 | 0.52 | 200-218 |
| 30 | CUPVSiirt265 | 2 | 1.78 | 0.13 | 0.44 | 0.34 | 194-198 |
| 31 | CUPVSiirt271 | 5 | 3.37 | 0.78 | 0.70 | 0.65 | 98-114 |
| 32 | CUPVSiirt284 | 3 | 2.18 | 0.42 | 0.54 | 0.46 | 220-243 |
| 33 | CUPVSiirt294 | 5 | 2.65 | 0.38 | 0.62 | 0.56 | 117-128 |
| 34 | CUPVSiirt297 | 4 | 2.42 | 0.50 | 0.59 | 0.50 | 136-146 |
| 35 | CUPVSiirt298 | 6 | 3.40 | 0.71 | 0.71 | 0.67 | 121-133 |
| 36 | CUPVSiirt308 | 5 | 3.10 | 0.35 | 0.68 | 0.62 | 167-175 |
| 37 | CUPVSiirt312 | 2 | 1.18 | 0.17 | 0.15 | 0.14 | 164-166 |
| 38 | CUPVSiirt316 | 5 | 2.70 | 0.38 | 0.63 | 0.59 | 254-280 |
| 39 | CUPVSiirt320 | 2 | 1.97 | 0.88 | 0.49 | 0.37 | 173-179 |
| 40 | CUPVSiirt328 | 4 | 2.00 | 0.41 | 0.50 | 0.47 | 186-196 |
| 41 | CUPVSiirt333 | 4 | 2.19 | 0.32 | 0.54 | 0.46 | 144-150 |
| 42 | CUPVSiirt340 | 6 | 3.36 | 0.58 | 0.70 | 0.66 | 129-163 |
| 43 | CUPVSiirt343 | 4 | 1.96 | 0.33 | 0.49 | 0.43 | 121-127 |
| 44 | CUPVSiirt349 | 4 | 3.59 | 0.58 | 0.72 | 0.67 | 174-190 |
| 45 | CUPVSiirt357 | 9 | 5.60 | 0.77 | 0.82 | 0.80 | 193-231 |
| 46 | CUPVSiirt358 | 9 | 5.80 | 0.27 | 0.83 | 0.81 | 215-269 |
| 47 | CUPVSiirt368 | 3 | 1.40 | 0.05 | 0.28 | 0.26 | 153-161 |
| 48 | CUPVSiirt415 | 3 | 2.43 | 0.30 | 0.59 | 0.52 | 158-170 |
| 49 | CUPVSiirt436 | 5 | 2.32 | 0.83 | 0.57 | 0.51 | 92-110 |
| 50 | CUPVSiirt446 | 2 | 1.99 | 0.42 | 0.50 | 0.37 | 236-242 |
| 51 | CUPVSiirt465 | 9 | 5.12 | 0.54 | 0.80 | 0.78 | 143-167 |
| 52 | CUPVSiirt472 | 3 | 1.79 | 0.42 | 0.44 | 0.38 | 172-185 |
| 53 | CUPVSiirt476 | 4 | 2.59 | 0.58 | 0.61 | 0.57 | 162-171 |
| 54 | CUPVSiirt479 | 5 | 2.13 | 0.61 | 0.53 | 0.49 | 165-192 |
| 55 | CUPVSiirt496 | 4 | 1.69 | 0.32 | 0.41 | 0.37 | 170-177 |
| 56 | CUPVSiirt501 | 3 | 1.58 | 0.29 | 0.37 | 0.32 | 145-158 |
| 57 | CUPVSiirt505 | 2 | 1.55 | 0.29 | 0.35 | 0.29 | 167-173 |
| 58 | CUPVSiirt509 | 2 | 1.25 | 0.23 | 0.20 | 0.18 | 170-180 |
| 59 | CUPVSiirt543 | 4 | 3.03 | 0.71 | 0.67 | 0.61 | 136-145 |
| 60 | CUPVSiirt565 | 6 | 3.14 | 0.41 | 0.68 | 0.64 | 151-164 |
| 61 | CUPVSiirt568x | 3 | 1.29 | 0.17 | 0.23 | 0.21 | 100-103 |
| 62 | CUPVSiirt568y | 10 | 4.77 | 0.55 | 0.79 | 0.77 | 121-145 |
| 63 | CUPVSiirt569 | 3 | 2.23 | 0.83 | 0.55 | 0.48 | 101-109 |
| 64 | CUPVSiirt598 | 5 | 4.19 | 0.63 | 0.76 | 0.72 | 169-190 |
| 65 | CUPVSiirt600 | 4 | 2.44 | 0.63 | 0.59 | 0.50 | 229-241 |
| 66 | CUPVSiirt616 | 9 | 7.6 | 0.71 | 0.87 | 0.85 | 172-204 |
| 67 | CUPVSiirt617 | 3 | 2.17 | 0.50 | 0.54 | 0.43 | 277-290 |
| 68 | CUPVSiirt621 | 5 | 1.64 | 0.42 | 0.39 | 0.37 | 93-129 |
| 69 | CUPVSiirt625 | 7 | 4.25 | 0.41 | 0.76 | 0.73 | 159-175 |
| 70 | CUPVSiirt649 | 2 | 1.98 | 0.48 | 0.50 | 0.37 | 158-165 |
| 71 | CUPVSiirt660 | 3 | 1.94 | 0.58 | 0.48 | 0.39 | 135-139 |
| 72 | CUPVSiirt661 | 4 | 1.69 | 0.32 | 0.41 | 0.37 | 253-260 |
| 73 | CUPVSiirt674 | 3 | 1.69 | 0.32 | 0.41 | 0.34 | 245-249 |
| 74 | CUPVSiirt689x | 4 | 2.07 | 0.43 | 0.52 | 0.48 | 200-208 |
| 75 | CUPVSiirt689y | 4 | 2.49 | 0.55 | 0.60 | 0.51 | 306-314 |
| 76 | CUPVSiirt690 | 2 | 1.55 | 0.21 | 0.35 | 0.29 | 215-221 |
| 77 | CUPVSiirt712 | 3 | 2.18 | 0.50 | 0.54 | 0.46 | 182-192 |
| 78 | CUPVSiirt715 | 3 | 1.18 | 0.17 | 0.16 | 0.15 | 158-170 |
| 79 | CUPVSiirt719 | 6 | 3.16 | 0.87 | 0.68 | 0.65 | 206-221 |
| 80 | CUPVSiirt724 | 2 | 1.80 | 0.42 | 0.44 | 0.35 | 135-138 |
| 81 | CUPVSiirt743 | 7 | 3.27 | 0.57 | 0.69 | 0.65 | 181-205 |
| 82 | CUPVSiirt764 | 6 | 3.33 | 0.83 | 0.70 | 0.66 | 164-206 |
| 83 | CUPVSiirt768 | 3 | 1.95 | 0.67 | 0.49 | 0.42 | 206-220 |
| 84 | CUPVSiirt782 | 6 | 4.37 | 0.30 | 0.77 | 0.74 | 172-197 |
| 85 | CUPVSiirt788 | 5 | 2.85 | 0.38 | 0.65 | 0.59 | 222-238 |
| 86 | CUPVSiirt794 | 5 | 2.98 | 0.67 | 0.66 | 0.63 | 216-244 |
| 87 | CUPVSiirt796 | 5 | 3.87 | 0.62 | 0.74 | 0.70 | 104-130 |
| 88 | CUPVSiirt803 | 4 | 2.05 | 0.29 | 0.51 | 0.47 | 202-106 |
| 89 | CUPVSiirt818 | 7 | 3.07 | 0.50 | 0.67 | 0.64 | 171-185 |
| 90 | CUPVSiirt838 | 5 | 2.60 | 0.86 | 0.61 | 0.55 | 159-181 |
| 91 | CUPVSiirt841 | 6 | 3.14 | 0.48 | 0.68 | 0.64 | 163-186 |
| 92 | CUPVSiirt847 | 7 | 4.26 | 0.62 | 0.77 | 0.73 | 226-256 |
| 93 | CUPVSiirt855 | 6 | 3.27 | 0.70 | 0.69 | 0.66 | 248-274 |
| 94 | CUPVSiirt858 | 7 | 3.81 | 0.85 | 0.74 | 0.70 | 167-198 |
| 95 | CUPVSiirt875 | 3 | 2.03 | 0.17 | 0.51 | 0.42 | 157-165 |
| 96 | CUPVSiirt876 | 7 | 4.80 | 0.96 | 0.79 | 0.76 | 185-207 |
| 97 | CUPVSiirt883 | 5 | 1.89 | 0.29 | 0.47 | 0.44 | 172-188 |
| 98 | CUPVSiirt889 | 6 | 3.02 | 0.58 | 0.67 | 0.61 | 168-188 |
| 99 | CUPVSiirt891 | 3 | 1.83 | 0.30 | 0.45 | 0.37 | 142-161 |
| 100 | CUPVSiirt907 | 3 | 2.36 | 0.57 | 0.58 | 0.48 | 92-98 |
| 101 | CUPVSiirt929 | 6 | 2.26 | 0.46 | 0.56 | 0.52 | 85-101 |
| 102 | CUPVSiirt931 | 5 | 2.61 | 0.21 | 0.62 | 0.55 | 146-160 |
| 103 | CUPVSiirt932 | 7 | 4.25 | 0.65 | 0.76 | 0.73 | 123-183 |
| 104 | CUPVSiirt949 | 5 | 1.97 | 0.38 | 0.49 | 0.46 | 160-197 |
| 105 | CUPVSiirt951 | 2 | 1.13 | 0.13 | 0.12 | 0.11 | 164-167 |
| 106 | CUPVSiirt956 | 10 | 2.63 | 0.43 | 0.62 | 0.60 | 133-200 |
| 107 | CUPVSiirt961 | 5 | 2.51 | 0.21 | 0.60 | 0.54 | 201-210 |
| 108 | CUPVSiirt975 | 3 | 1.61 | 0.38 | 0.38 | 0.34 | 145-154 |
| 109 | CUPVSiirt986 | 3 | 2.8 | 0.46 | 0.64 | 0.57 | 157-169 |
| 110 | CUPVSiirt989 | 5 | 1.89 | 0.33 | 0.47 | 0.44 | 148-176 |
| 111 | CUPVSiirt1003 | 4 | 3.02 | 0.55 | 0.67 | 0.61 | 81-112 |
| 112 | CUPVSiirt1008 | 4 | 1.87 | 0.35 | 0.47 | 0.42 | 165-174 |
| 113 | CUPVSiirt1017 | 6 | 2.43 | 0.42 | 0.59 | 0.54 | 218-236 |
| 114 | CUPVSiirt1021 | 3 | 2.23 | 0.71 | 0.55 | 0.47 | 127-135 |
| 115 | CUPVSiirt1041 | 4 | 2.61 | 0.58 | 0.62 | 0.55 | 107-114 |
| 116 | CUPVSiirt1043 | 3 | 1.23 | 0.21 | 0.19 | 0.18 | 104-134 |
| 117 | CUPVSiirt1047 | 3 | 1.40 | 0.00 | 0.29 | 0.26 | 140-148 |
| 118 | CUPVSiirt1053 | 2 | 1.84 | 0.38 | 0.46 | 0.35 | 159-165 |
| 119 | CUPVSiirt1055 | 2 | 1.18 | 0.17 | 0.15 | 0.14 | 155-169 |
| 120 | CUPVSiirt1057 | 6 | 3.33 | 0.50 | 0.70 | 0.65 | 237-254 |
| 121 | CUPVSiirt1062 | 3 | 2.18 | 0.50 | 0.54 | 0.46 | 139-144 |
| 122 | CUPVSiirt1071 | 6 | 2.91 | 0.55 | 0.66 | 0.59 | 146-169 |
| 123 | CUPVSiirt1092 | 4 | 3.12 | 0.75 | 0.68 | 0.62 | 149-155 |
| 124 | CUPVSiirt1095 | 7 | 5.28 | 0.88 | 0.81 | 0.79 | 248-290 |
| 125 | CUPVSiirt1116 | 6 | 2.13 | 0.54 | 0.53 | 0.49 | 158-191 |
| 126 | CUPVSiirt1117 | 8 | 4.13 | 0.71 | 0.76 | 0.72 | 158-172 |
| 127 | CUPVSiirt1120 | 5 | 2.02 | 0.58 | 0.50 | 0.46 | 198-208 |
| 128 | CUPVSiirt1122 | 4 | 3.42 | 0.54 | 0.71 | 0.66 | 208-214 |
| 129 | CUPVSiirt1127 | 6 | 2.37 | 0.54 | 0.58 | 0.55 | 120-134 |
| 130 | CUPVSiirt1140 | 4 | 2.71 | 0.35 | 0.63 | 0.56 | 162-176 |
| 131 | CUPVSiirt1145 | 5 | 2.81 | 0.57 | 0.64 | 0.59 | 158-173 |
| 132 | CUPVSiirt1153 | 3 | 2.07 | 0.08 | 0.52 | 0.42 | 194-201 |
| 133 | CUPVSiirt1163 | 5 | 3.24 | 0.68 | 0.69 | 0.64 | 156-166 |
| 134 | CUPVSiirt1171 | 2 | 1.14 | 0.13 | 0.12 | 0.11 | 251-252 |
| 135 | CUPVSiirt1182 | 4 | 2.32 | 0.54 | 0.57 | 0.52 | 167-179 |
| 136 | CUPVSiirt1183 | 2 | 1.91 | 0.52 | 0.48 | 0.36 | 244-250 |
| 137 | CUPVSiirt1188 | 2 | 1.57 | 0.48 | 0.36 | 0.30 | 158-168 |
| 138 | CUPVSiirt1189 | 3 | 1.94 | 0.38 | 0.48 | 0.40 | 259-267 |
| 139 | CUPVSiirt1191 | 4 | 3.07 | 0.63 | 0.67 | 0.63 | 158-190 |
| 140 | CUPVSiirt1202 | 5 | 2.32 | 0.29 | 0.57 | 0.52 | 176-192 |
| 141 | CUPVSiirt1214 | 3 | 1.47 | 0.29 | 0.32 | 0.29 | 160-164 |
| 142 | CUPVSiirt1224 | 5 | 4.32 | 0.80 | 0.77 | 0.73 | 257-275 |
| 143 | CUPVSiirt1238 | 3 | 1.53 | 0.42 | 0.35 | 0.32 | 239-252 |
| 144 | CUPVSiirt1243 | 6 | 2.99 | 0.26 | 0.67 | 0.61 | 144-156 |
| 145 | CUPVSiirt1250 | 6 | 2.53 | 0.54 | 0.60 | 0.55 | 175-189 |
| 146 | CUPVSiirt1260 | 4 | 1.29 | 0.25 | 0.23 | 0.21 | 166-175 |
| 147 | CUPVSiirt1267 | 3 | 2.06 | 0.57 | 0.52 | 0.44 | 137-140 |
| 148 | CUPVSiirt1271 | 4 | 1.30 | 0.25 | 0.23 | 0.22 | 223-232 |
| 149 | CUPVSiirt1273 | 2 | 2.00 | 1.00 | 0.50 | 0.38 | 140-160 |
| 150 | CUPVSiirt1278 | 2 | 1.55 | 0.13 | 0.35 | 0.29 | 176-181 |
| 151 | CUPVSiirt1322 | 5 | 2.00 | 0.55 | 0.50 | 0.47 | 218-237 |
| 152 | CUPVSiirt1326 | 5 | 3.26 | 0.55 | 0.69 | 0.65 | 189-198 |
| 153 | CUPVSiirt1330 | 11 | 6.26 | 0.74 | 0.84 | 0.82 | 159-185 |
| 154 | CUPVSiirt1331 | 3 | 1.90 | 0.38 | 0.47 | 0.42 | 104-115 |
| 155 | CUPVSiirt1345 | 2 | 1.65 | 0.38 | 0.39 | 0.32 | 185-191 |
| 156 | CUPVSiirt1353 | 8 | 2.78 | 0.73 | 0.64 | 0.62 | 173-197 |
| 157 | CUPVSiirt1360 | 5 | 3.32 | 0.46 | 0.70 | 0.66 | 127-135 |
| 158 | CUPVSiirt1372 | 5 | 1.92 | 0.09 | 0.48 | 0.45 | 182-194 |
| 159 | CUPVSiirt1378 | 3 | 2.80 | 0.21 | 0.64 | 0.57 | 95-97 |
| 160 | CUPVSiirt1388 | 3 | 2.28 | 0.65 | 0.56 | 0.47 | 183-201 |
| 161 | CUPVSiirt1394 | 4 | 2.31 | 0.38 | 0.57 | 0.49 | 246-266 |
| 162 | CUPVSiirt1399 | 2 | 1.09 | 0.08 | 0.08 | 0.08 | 183-193 |
| 163 | CUPVSiirt1400 | 3 | 2.48 | 0.61 | 0.60 | 0.51 | 158-168 |
| 164 | CUPVSiirt1402 | 4 | 2.83 | 0.54 | 0.65 | 0.60 | 178-187 |
| 165 | CUPVSiirt1405 | 6 | 3.72 | 0.58 | 0.73 | 0.69 | 179-211 |
| 166 | CUPVSiirt1406 | 3 | 2.07 | 0.17 | 0.52 | 0.44 | 172-191 |
| 167 | CUPVSiirt1413 | 2 | 1.98 | 0.39 | 0.50 | 0.37 | 172-176 |
| 168 | CUPVSiirt1417 | 6 | 2.36 | 0.43 | 0.58 | 0.55 | 138-176 |
| 169 | CUPVSiirt1418 | 10 | 3.45 | 0.52 | 0.71 | 0.68 | 131-159 |
| 170 | CUPVSiirt1431 | 3 | 1.60 | 0.18 | 0.38 | 0.34 | 209-215 |
| 171 | CUPVSiirt1438 | 4 | 1.87 | 0.13 | 0.46 | 0.43 | 271-280 |
| 172 | CUPVSiirt1442 | 4 | 1.42 | 0.24 | 0.29 | 0.28 | 110-118 |
| 173 | CUPVSiirt1457 | 5 | 3.95 | 0.46 | 0.75 | 0.71 | 157-181 |
| 174 | CUPVSiirt1477 | 5 | 2.59 | 0.48 | 0.61 | 0.57 | 109-126 |
| 175 | CUPVSiirt1478 | 8 | 4.04 | 0.54 | 0.75 | 0.72 | 92-119 |
| 176 | CUPVSiirt1517 | 5 | 1.94 | 0.22 | 0.48 | 0.45 | 213-225 |
| 177 | CUPVSiirt1547 | 2 | 1.04 | 0.04 | 0.04 | 0.04 | 120-126 |
| 178 | CUPVSiirt1564 | 5 | 3.26 | 0.26 | 0.69 | 0.63 | 211-218 |
| 179 | CUPVSiirt1567 | 5 | 2.67 | 0.58 | 0.63 | 0.58 | 195-203 |
| 180 | CUPVSiirt1599 | 6 | 4.00 | 0.55 | 0.75 | 0.72 | 320-345 |
| 181 | CUPVSiirt1611 | 2 | 1.18 | 0.17 | 0.15 | 0.14 | 194-205 |
| 182 | CUPVSiirt1626 | 4 | 2.78 | 0.45 | 0.64 | 0.57 | 134-144 |
| 183 | CUPVSiirt1628 | 4 | 1.87 | 0.42 | 0.46 | 0.43 | 125-146 |
| 184 | CUPVSiirt1639 | 4 | 2.67 | 0.46 | 0.63 | 0.57 | 168-194 |
| 185 | CUPVSiirt1640 | 5 | 1.63 | 0.13 | 0.39 | 0.36 | 161-176 |
| 186 | CUPVSiirt1652 | 7 | 2.92 | 0.75 | 0.66 | 0.62 | 174-193 |
| 187 | CUPVSiirt1655 | 3 | 2.28 | 0.57 | 0.56 | 0.47 | 150-156 |
| 188 | CUPVSiirt1658 | 2 | 1.35 | 0.30 | 0.26 | 0.22 | 130-136 |
| 189 | CUPVSiirt1667 | 5 | 2.26 | 0.45 | 0.56 | 0.49 | 170-186 |
| 190 | CUPVSiirt1688 | 5 | 3.18 | 0.78 | 0.69 | 0.63 | 168-175 |
| 191 | CUPVSiirt1734 | 4 | 2.19 | 0.54 | 0.54 | 0.49 | 168-178 |
| 192 | CUPVSiirt1740 | 3 | 2.34 | 0.46 | 0.57 | 0.51 | 162-174 |
| 193 | CUPVSiirt1742 | 7 | 2.94 | 0.41 | 0.66 | 0.62 | 181-213 |
| 194 | CUPVSiirt1749 | 4 | 2.68 | 0.57 | 0.63 | 0.55 | 145-164 |
| 195 | CUPVSiirt1759 | 4 | 3.39 | 0.58 | 0.70 | 0.65 | 139-159 |
| 196 | CUPVSiirt1764 | 5 | 2.50 | 0.54 | 0.60 | 0.56 | 154-178 |
| 197 | CUPVSiirt1768 | 4 | 1.66 | 0.48 | 0.40 | 0.37 | 121-128 |
| 198 | CUPVSiirt1784 | 4 | 3.13 | 0.58 | 0.68 | 0.62 | 194-202 |
| 199 | CUPVSiirt1788 | 2 | 1.18 | 0.17 | 0.15 | 0.14 | 183-189 |
| 200 | CUPVSiirt1797 | 3 | 1.79 | 0.54 | 0.44 | 0.36 | 155-169 |
|  | Total | 897 | - | - | - | - |  |
|  | Mean | 4.5 | 2.57 | 0.46 | 0.55 | 0.50 |  |
