# Supplementary material for: Genome survey of pistachio (Pistacia vera L.) by next generation sequencing: Development of novel SSR markers and genetic diversity in Pistacia species
Source: BMC Genomics. 2016 Dec 7;17:998. doi: 10.1186/s12864-016-3359-x (PMC5142174; doi:10.1186/s12864-016-3359-x)
Supplement: Additional file 3: — Genetic diversity measures in P. atlantica: allele ranges, number of alleles (Na), number of effective alleles (Ne), observed heterozygosity (Ho), expected heterozygosity (He), and PIC values of 161 polymorphic SSR loci. (DOCX 37 kb) [file 12864_2016_3359_MOESM3_ESM.docx]

**Additional file 3. Number of alleles (Na), Number of effective alleles (Ne), observed heterozygosity (Ho), expected heterozygosity (He), PIC values and allele range of 161 polymorphic SSR loci developed from *Pistacia atlantica.***

| **No** | **Loci** | **Na** | **Ne** | **Ho** | **He** | **PIC** | **Allele Range**  **(bp)** |
| --- | --- | --- | --- | --- | --- | --- | --- |
| 1 | CUPVSiirt15 | 3 | 2.46 | 0.50 | 0.59 | 0.51 | 104-108 |
| 2 | CUPVSiirt22 | 3 | 2.46 | 0.25 | 0.59 | 0.51 | 161-174 |
| 3 | CUPVSiirt26 | 2 | 1.28 | 0.25 | 0.22 | 0.19 | 176-188 |
| 4 | CUPVSiirt37 | 4 | 2.91 | 0.75 | 0.66 | 0.60 | 140-163 |
| 5 | CUPVSiirt71 | 2 | 2.00 | 0.00 | 0.50 | 0.38 | 164-166 |
| 6 | CUPVSiirt76 | 3 | 2.13 | 0.75 | 0.53 | 0.47 | 170-176 |
| 7 | CUPVSiirt86 | 4 | 3.56 | 1.00 | 0.72 | 0.67 | 128-155 |
| 8 | CUPVSiirt115 | 2 | 1.60 | 0.50 | 0.38 | 0.30 | 166-171 |
| 9 | CUPVSiirt129 | 5 | 4.00 | 1.00 | 0.75 | 0.71 | 147-172 |
| 10 | CUPVSiirt131 | 2 | 1.60 | 0.50 | 0.38 | 0.30 | 163-169 |
| 11 | CUPVSiirt140 | 2 | 1.60 | 0.50 | 0.38 | 0.30 | 229-231 |
| 12 | CUPVSiirt149 | 5 | 4.00 | 0.50 | 0.75 | 0.71 | 105-136 |
| 13 | CUPVSiirt151 | 2 | 1.28 | 0.25 | 0.22 | 0.19 | 154-162 |
| 14 | CUPVSiirt158 | 2 | 1.28 | 0.25 | 0.22 | 0.19 | 221-229 |
| 15 | CUPVSiirt186 | 5 | 4.00 | 0.75 | 0.75 | 0.71 | 140-179 |
| 16 | CUPVSiirt230 | 3 | 2.67 | 0.00 | 0.63 | 0.55 | 191-216 |
| 17 | CUPVSiirt243 | 5 | 3.20 | 0.50 | 0.69 | 0.65 | 142-149 |
| 18 | CUPVSiirt256 | 3 | 1.68 | 0.25 | 0.41 | 0.37 | 177-199 |
| 19 | CUPVSiirt259 | 3 | 2.46 | 0.50 | 0.59 | 0.51 | 186-195 |
| 20 | CUPVSiirt265 | 2 | 1.28 | 0.25 | 0.22 | 0.19 | 194-196 |
| 21 | CUPVSiirt271 | 3 | 1.68 | 0.50 | 0.41 | 0.37 | 102-110 |
| 22 | CUPVSiirt284 | 2 | 1.60 | 0.00 | 0.38 | 0.30 | 229-235 |
| 23 | CUPVSiirt294 | 2 | 1.60 | 0.00 | 0.38 | 0.30 | 124-138 |
| 24 | CUPVSiirt298 | 4 | 2.91 | 0.25 | 0.66 | 0.60 | 154-166 |
| 25 | CUPVSiirt308 | 3 | 1.68 | 0.50 | 0.41 | 0.37 | 166-168 |
| 26 | CUPVSiirt312 | 3 | 2.13 | 0.75 | 0.53 | 0.47 | 154-172 |
| 27 | CUPVSiirt316 | 4 | 2.91 | 0.25 | 0.66 | 0.60 | 259-280 |
| 28 | CUPVSiirt328 | 3 | 2.00 | 0.33 | 0.50 | 0.45 | 181-204 |
| 29 | CUPVSiirt333 | 4 | 2.91 | 1.00 | 0.66 | 0.60 | 144-153 |
| 30 | CUPVSiirt340 | 5 | 4.00 | 0.50 | 0.75 | 0.71 | 129-171 |
| 31 | CUPVSiirt349 | 7 | 6.40 | 0.75 | 0.84 | 0.82 | 172-199 |
| 32 | CUPVSiirt357 | 3 | 2.13 | 0.50 | 0.53 | 0.47 | 193-202 |
| 33 | CUPVSiirt368 | 3 | 2.67 | 0.00 | 0.63 | 0.55 | 166-188 |
| 34 | CUPVSiirt415 | 2 | 1.28 | 0.25 | 0.22 | 0.19 | 145-153 |
| 35 | CUPVSiirt436 | 4 | 3.56 | 0.25 | 0.72 | 0.67 | 92-114 |
| 36 | CUPVSiirt446 | 3 | 2.13 | 0.25 | 0.53 | 0.47 | 230-242 |
| 37 | CUPVSiirt465 | 6 | 5.33 | 0.50 | 0.81 | 0.79 | 145-178 |
| 38 | CUPVSiirt472 | 3 | 1.68 | 0.25 | 0.41 | 0.37 | 294-325 |
| 39 | CUPVSiirt476 | 2 | 1.28 | 0.25 | 0.22 | 0.19 | 157-162 |
| 40 | CUPVSiirt479 | 2 | 1.38 | 0.33 | 0.28 | 0.24 | 165-187 |
| 41 | CUPVSiirt496 | 2 | 1.60 | 0.00 | 0.38 | 0.30 | 181-199 |
| 42 | CUPVSiirt505 | 2 | 1.60 | 0.50 | 0.38 | 0.30 | 175-179 |
| 43 | CUPVSiirt509 | 4 | 2.29 | 0.50 | 0.56 | 0.52 | 162-181 |
| 44 | CUPVSiirt543 | 4 | 2.91 | 0.75 | 0.66 | 0.60 | 132-142 |
| 45 | CUPVSiirt565 | 2 | 1.28 | 0.25 | 0.22 | 0.19 | 140-142 |
| 46 | CUPVSiirt568y | 4 | 3.60 | 0.70 | 0.70 | 0.67 | 123-126 |
| 47 | CUPVSiirt569 | 3 | 2.67 | 1.00 | 0.63 | 0.55 | 95-102 |
| 48 | CUPVSiirt598 | 4 | 2.29 | 0.75 | 0.56 | 0.52 | 163-186 |
| 49 | CUPVSiirt600 | 2 | 1.88 | 0.75 | 0.47 | 0.36 | 229-231 |
| 50 | CUPVSiirt616 | 4 | 2.91 | 0.75 | 0.66 | 0.6 | 176-204 |
| 51 | CUPVSiirt625 | 3 | 2.46 | 0.50 | 0.59 | 0.51 | 169-175 |
| 52 | CUPVSiirt660 | 3 | 2.46 | 0.50 | 0.59 | 0.51 | 131-135 |
| 53 | CUPVSiirt661 | 3 | 2.46 | 1.00 | 0.59 | 0.51 | 251-255 |
| 54 | CUPVSiirt674 | 3 | 1.68 | 0.50 | 0.41 | 0.37 | 235-243 |
| 55 | CUPVSiirt689x | 2 | 1.80 | 0.00 | 0.44 | 0.35 | 200-210 |
| 56 | CUPVSiirt689y | 2 | 1.88 | 0.75 | 0.47 | 0.36 | 306-314 |
| 57 | CUPVSiirt712 | 5 | 4.00 | 1.00 | 0.75 | 0.71 | 180-198 |
| 58 | CUPVSiirt715 | 4 | 2.29 | 0.50 | 0.56 | 0.52 | 160-176 |
| 59 | CUPVSiirt719 | 4 | 3.20 | 1.00 | 0.69 | 0.63 | 209-231 |
| 60 | CUPVSiirt742 | 3 | 2.91 | 0.50 | 0.66 | 0.58 | 201-205 |
| 61 | CUPVSiirt743 | 3 | 1.68 | 0.25 | 0.41 | 0.37 | 163-167 |
| 62 | CUPVSiirt764 | 2 | 1.88 | 0.25 | 0.47 | 0.36 | 164-176 |
| 63 | CUPVSiirt768 | 3 | 2.46 | 1.00 | 0.59 | 0.51 | 206-218 |
| 64 | CUPVSiirt782 | 4 | 2.91 | 1.00 | 0.66 | 0.60 | 176-188 |
| 65 | CUPVSiirt788 | 3 | 2.46 | 0.50 | 0.59 | 0.51 | 214-220 |
| 66 | CUPVSiirt794 | 3 | 1.68 | 0.25 | 0.41 | 0.37 | 216-238 |
| 67 | CUPVSiirt796 | 4 | 2.91 | 0.50 | 0.66 | 0.60 | 114-127 |
| 68 | CUPVSiirt818 | 2 | 1.28 | 0.25 | 0.22 | 0.19 | 165-181 |
| 69 | CUPVSiirt836 | 3 | 2.67 | 0.00 | 0.63 | 0.55 | 164-166 |
| 70 | CUPVSiirt838 | 5 | 4.57 | 1.00 | 0.78 | 0.75 | 159-171 |
| 71 | CUPVSiirt841 | 7 | 6.40 | 1.00 | 0.84 | 0.82 | 155-185 |
| 72 | CUPVSiirt858 | 2 | 1.28 | 0.25 | 0.22 | 0.19 | 177-183 |
| 73 | CUPVSiirt875 | 5 | 4.00 | 0.75 | 0.75 | 0.71 | 157-177 |
| 74 | CUPVSiirt876 | 3 | 2.46 | 0.50 | 0.59 | 0.51 | 183-187 |
| 75 | CUPVSiirt891 | 2 | 1.28 | 0.25 | 0.22 | 0.19 | 142-148 |
| 76 | CUPVSiirt907 | 2 | 1.28 | 0.25 | 0.22 | 0.19 | 92-95 |
| 77 | CUPVSiirt924 | 2 | 1.60 | 0.00 | 0.38 | 0.30 | 87-89 |
| 78 | CUPVSiirt929 | 4 | 2.91 | 0.25 | 0.66 | 0.60 | 87-105 |
| 79 | CUPVSiirt949 | 2 | 1.88 | 0.25 | 0.47 | 0.36 | 167-179 |
| 80 | CUPVSiirt956 | 4 | 3.60 | 0.67 | 0.72 | 0.67 | 162-198 |
| 81 | CUPVSiirt961 | 3 | 2.46 | 0.50 | 0.59 | 0.51 | 190-204 |
| 82 | CUPVSiirt989 | 2 | 1.60 | 0.00 | 0.38 | 0.30 | 148-152 |
| 83 | CUPVSiirt1003 | 3 | 2.00 | 0.33 | 0.50 | 0.45 | 81-102 |
| 84 | CUPVSiirt1017 | 4 | 2.91 | 1.00 | 0.66 | 0.60 | 214-245 |
| 85 | CUPVSiirt1021 | 2 | 1.88 | 0.75 | 0.47 | 0.36 | 125-131 |
| 86 | CUPVSiirt1043 | 4 | 3.20 | 0.75 | 0.69 | 0.63 | 113-147 |
| 87 | CUPVSiirt1047 | 3 | 2.67 | 1.00 | 0.63 | 0.55 | 127-157 |
| 88 | CUPVSiirt1053 | 3 | 2.13 | 0.25 | 0.53 | 0.47 | 178-189 |
| 89 | CUPVSiirt1055 | 3 | 2.91 | 0.50 | 0.66 | 0.58 | 155-169 |
| 90 | CUPVSiirt1057 | 5 | 4.00 | 0.75 | 0.75 | 0.71 | 230-258 |
| 91 | CUPVSiirt1062 | 5 | 4.00 | 0.75 | 0.75 | 0.71 | 138-146 |
| 92 | CUPVSiirt1071 | 2 | 1.60 | 0.50 | 0.38 | 0.30 | 146-150 |
| 93 | CUPVSiirt1092 | 6 | 5.33 | 1.00 | 0.81 | 0.79 | 145-163 |
| 94 | CUPVSiirt1095 | 2 | 2.00 | 0.33 | 0.50 | 0.38 | 242-244 |
| 95 | CUPVSiirt1116 | 5 | 3.20 | 0.75 | 0.69 | 0.65 | 151-170 |
| 96 | CUPVSiirt1117 | 3 | 2.13 | 0.75 | 0.53 | 0.47 | 150-170 |
| 97 | CUPVSiirt1120 | 3 | 2.13 | 0.50 | 0.53 | 0.47 | 198-218 |
| 98 | CUPVSiirt1122 | 2 | 1.60 | 0.00 | 0.38 | 0.30 | 209-215 |
| 99 | CUPVSiirt1140 | 2 | 1.88 | 0.25 | 0.47 | 0.36 | 162-166 |
| 100 | CUPVSiirt1145 | 3 | 2.13 | 0.75 | 0.53 | 0.47 | 162-174 |
| 101 | CUPVSiirt1153 | 2 | 1.88 | 0.25 | 0.47 | 0.36 | 192-195 |
| 102 | CUPVSiirt1171 | 5 | 4.00 | 0.75 | 0.75 | 0.71 | 241-270 |
| 103 | CUPVSiirt1182 | 4 | 2.91 | 0.75 | 0.66 | 0.60 | 161-173 |
| 104 | CUPVSiirt1188 | 2 | 1.88 | 0.25 | 0.47 | 0.36 | 158-168 |
| 105 | CUPVSiirt1189 | 2 | 1.88 | 0.25 | 0.47 | 0.36 | 247-249 |
| 106 | CUPVSiirt1191 | 2 | 2.00 | 0.00 | 0.50 | 0.38 | 162-164 |
| 107 | CUPVSiirt1202 | 4 | 2.29 | 0.50 | 0.56 | 0.52 | 174-182 |
| 108 | CUPVSiirt1214 | 4 | 4.00 | 0.00 | 0.75 | 0.70 | 160-171 |
| 109 | CUPVSiirt1224 | 3 | 2.13 | 0.25 | 0.53 | 0.47 | 257-265 |
| 110 | CUPVSiirt1238 | 3 | 2.67 | 0.00 | 0.63 | 0.55 | 239-249 |
| 111 | CUPVSiirt1243 | 2 | 1.60 | 0.00 | 0.38 | 0.30 | 138-142 |
| 112 | CUPVSiirt1250 | 2 | 1.60 | 0.00 | 0.38 | 0.30 | 176-177 |
| 113 | CUPVSiirt1260 | 4 | 2.91 | 0.75 | 0.66 | 0.60 | 157-172 |
| 114 | CUPVSiirt1267 | 3 | 1.68 | 0.50 | 0.41 | 0.37 | 133-155 |
| 115 | CUPVSiirt1271 | 2 | 1.60 | 0.50 | 0.38 | 0.30 | 221-223 |
| 116 | CUPVSiirt1273 | 3 | 1.68 | 0.50 | 0.41 | 0.37 | 140-154 |
| 117 | CUPVSiirt1322 | 3 | 2.13 | 0.75 | 0.53 | 0.47 | 218-223 |
| 118 | CUPVSiirt1326 | 5 | 4.57 | 1.00 | 0.78 | 0.75 | 192-211 |
| 119 | CUPVSiirt1330 | 4 | 2.91 | 0.75 | 0.66 | 0.60 | 157-183 |
| 120 | CUPVSiirt1331 | 5 | 4.57 | 0.75 | 0.78 | 0.75 | 110-119 |
| 121 | CUPVSiirt1345 | 2 | 1.38 | 0.33 | 0.28 | 0.24 | 173-179 |
| 122 | CUPVSiirt1372 | 3 | 2.13 | 0.50 | 0.53 | 0.47 | 112-130 |
| 123 | CUPVSiirt1378 | 3 | 1.68 | 0.25 | 0.41 | 0.37 | 83-97 |
| 124 | CUPVSiirt1394 | 4 | 3.60 | 0.33 | 0.72 | 0.67 | 234-270 |
| 125 | CUPVSiirt1399 | 4 | 2.91 | 1.00 | 0.66 | 0.60 | 193-216 |
| 126 | CUPVSiirt1400 | 7 | 6.40 | 1.00 | 0.84 | 0.82 | 168-186 |
| 127 | CUPVSiirt1402 | 3 | 2.13 | 0.25 | 0.53 | 0.47 | 174-181 |
| 128 | CUPVSiirt1405 | 5 | 4.57 | 1.00 | 0.78 | 0.75 | 176-206 |
| 129 | CUPVSiirt1406 | 4 | 4.00 | 0.00 | 0.75 | 0.70 | 172-185 |
| 130 | CUPVSiirt1413 | 3 | 1.68 | 0.50 | 0.41 | 0.37 | 166-172 |
| 131 | CUPVSiirt1417 | 4 | 2.91 | 0.50 | 0.66 | 0.60 | 142-153 |
| 132 | CUPVSiirt1418 | 2 | 1.88 | 0.25 | 0.47 | 0.36 | 155-157 |
| 133 | CUPVSiirt1431 | 3 | 2.91 | 0.75 | 0.66 | 0.58 | 203-213 |
| 134 | CUPVSiirt1438 | 4 | 2.91 | 0.50 | 0.66 | 0.60 | 265-290 |
| 135 | CUPVSiirt1442 | 4 | 3.56 | 0.50 | 0.72 | 0.67 | 110-126 |
| 136 | CUPVSiirt1457 | 3 | 2.67 | 0.00 | 0.63 | 0.55 | 159-195 |
| 137 | CUPVSiirt1477 | 4 | 3.00 | 0.67 | 0.67 | 0.62 | 117-120 |
| 138 | CUPVSiirt1478 | 4 | 3.56 | 0.25 | 0.72 | 0.67 | 84-117 |
| 139 | CUPVSiirt1517 | 2 | 2.00 | 0.50 | 0.50 | 0.38 | 213-219 |
| 140 | CUPVSiirt1547 | 2 | 1.88 | 0.75 | 0.47 | 0.36 | 120-126 |
| 141 | CUPVSiirt1564 | 4 | 3.56 | 0.25 | 0.72 | 0.67 | 210-217 |
| 142 | CUPVSiirt1567 | 4 | 3.56 | 0.50 | 0.72 | 0.67 | 190-195 |
| 143 | CUPVSiirt1599 | 4 | 4.00 | 0.00 | 0.75 | 0.70 | 294-342 |
| 144 | CUPVSiirt1611 | 5 | 4.57 | 0.25 | 0.78 | 0.75 | 194-215 |
| 145 | CUPVSiirt1626 | 3 | 2.67 | 0.00 | 0.63 | 0.55 | 123-153 |
| 146 | CUPVSiirt1639 | 2 | 2.00 | 1.00 | 0.50 | 0.38 | 169-170 |
| 147 | CUPVSiirt1640 | 4 | 3.56 | 0.75 | 0.72 | 0.67 | 146-180 |
| 148 | CUPVSiirt1652 | 2 | 1.28 | 0.25 | 0.22 | 0.19 | 172-191 |
| 149 | CUPVSiirt1655 | 5 | 3.20 | 0.75 | 0.69 | 0.65 | 180-213 |
| 150 | CUPVSiirt1658 | 2 | 1.88 | 0.75 | 0.47 | 0.36 | 136-142 |
| 151 | CUPVSiirt1667 | 3 | 2.46 | 0.25 | 0.59 | 0.51 | 164-168 |
| 152 | CUPVSiirt1688 | 5 | 4.57 | 1.00 | 0.78 | 0.75 | 168-179 |
| 153 | CUPVSiirt1705 | 3 | 2.67 | 0.00 | 0.63 | 0.55 | 231-251 |
| 154 | CUPVSiirt1734 | 2 | 1.88 | 0.75 | 0.47 | 0.36 | 168-174 |
| 155 | CUPVSiirt1740 | 3 | 1.68 | 0.50 | 0.41 | 0.37 | 162-174 |
| 156 | CUPVSiirt1742 | 2 | 2.00 | 0.00 | 0.50 | 0.38 | 151-163 |
| 157 | CUPVSiirt1749 | 3 | 2.46 | 0.50 | 0.59 | 0.51 | 145-158 |
| 158 | CUPVSiirt1759 | 4 | 2.91 | 0.50 | 0.66 | 0.60 | 146-159 |
| 159 | CUPVSiirt1768 | 3 | 2.91 | 0.25 | 0.66 | 0.58 | 121-126 |
| 160 | CUPVSiirt1784 | 4 | 3.20 | 0.50 | 0.69 | 0.63 | 194-211 |
| 161 | CUPVSiirt1797 | 3 | 2.13 | 0.50 | 0.53 | 0.47 | 155-161 |
|  | Total | 527 |  |  |  |  |  |
|  | Mean | 3.3 | 2.59 | 0.48 | 0.56 | 0.49 |  |
