# Supplementary material for: Genome survey of pistachio (Pistacia vera L.) by next generation sequencing: Development of novel SSR markers and genetic diversity in Pistacia species
Source: BMC Genomics. 2016 Dec 7;17:998. doi: 10.1186/s12864-016-3359-x (PMC5142174; doi:10.1186/s12864-016-3359-x)
Supplement: Additional file 4: — Genetic diversity measures in P. integerrima: allele ranges, number of alleles (Na), number of effective alleles (Ne), observed heterozygosity (Ho), expected heterozygosity (He), and PIC values of 157 polymorphic SSR loci. (DOCX 37 kb) [file 12864_2016_3359_MOESM4_ESM.docx]

**Additional file 4. Number of alleles (Na), Number of effective alleles (Ne), observed heterozygosity (Ho), expected heterozygosity (He), PIC values and allele range of 157 polymorphic SSR loci developed from *Pistacia integerrima.***

| **No** | **Loci** | **Na** | **Ne** | **Ho** | **He** | **PIC** | **Allele range**  **(bp)** |
| --- | --- | --- | --- | --- | --- | --- | --- |
| 1 | CUPVSiirt15 | 3 | 2.13 | 0.25 | 0.53 | 0.47 | 104-123 |
| 2 | CUPVSiirt22 | 3 | 2.46 | 0.75 | 0.59 | 0.51 | 160-182 |
| 3 | CUPVSiirt26 | 2 | 1.88 | 0.75 | 0.47 | 0.36 | 164-188 |
| 4 | CUPVSiirt37 | 3 | 2.46 | 0.75 | 0.59 | 0.51 | 140-167 |
| 5 | CUPVSiirt50 | 2 | 1.38 | 0.33 | 0.28 | 0.24 | 162-170 |
| 6 | CUPVSiirt71 | 3 | 2.67 | 0.00 | 0.63 | 0.55 | 156-167 |
| 7 | CUPVSiirt76 | 3 | 2.13 | 0.50 | 0.53 | 0.47 | 172-176 |
| 8 | CUPVSiirt86 | 3 | 2.67 | 0.50 | 0.63 | 0.55 | 111-142 |
| 9 | CUPVSiirt115 | 2 | 1.88 | 0.25 | 0.47 | 0.36 | 166-171 |
| 10 | CUPVSiirt125 | 2 | 2.00 | 1.00 | 0.50 | 0.38 | 167-177 |
| 11 | CUPVSiirt129 | 4 | 3.56 | 1.00 | 0.72 | 0.67 | 135-172 |
| 12 | CUPVSiirt131 | 5 | 4.00 | 0.75 | 0.75 | 0.71 | 162-175 |
| 13 | CUPVSiirt149 | 3 | 2.13 | 0.75 | 0.53 | 0.47 | 104-108 |
| 14 | CUPVSiirt151 | 3 | 2.13 | 0.75 | 0.53 | 0.47 | 160-164 |
| 15 | CUPVSiirt171 | 3 | 2.13 | 0.75 | 0.53 | 0.47 | 142-161 |
| 16 | CUPVSiirt213 | 2 | 1.28 | 0.25 | 0.22 | 0.19 | 211-219 |
| 17 | CUPVSiirt238 | 4 | 2.91 | 0.50 | 0.66 | 0.60 | 171-191 |
| 18 | CUPVSiirt242 | 2 | 1.28 | 0.25 | 0.22 | 0.19 | 134-155 |
| 19 | CUPVSiirt243 | 3 | 2.91 | 0.75 | 0.66 | 0.58 | 140-149 |
| 20 | CUPVSiirt256 | 2 | 1.60 | 0.00 | 0.38 | 0.30 | 179-183 |
| 21 | CUPVSiirt259 | 3 | 2.46 | 1.00 | 0.59 | 0.51 | 186-210 |
| 22 | CUPVSiirt265 | 2 | 1.88 | 0.75 | 0.47 | 0.36 | 194-200 |
| 23 | CUPVSiirt271 | 2 | 2.00 | 0.00 | 0.50 | 0.38 | 101-104 |
| 24 | CUPVSiirt284 | 3 | 2.46 | 1.00 | 0.59 | 0.51 | 235-247 |
| 25 | CUPVSiirt294 | 3 | 1.68 | 0.50 | 0.41 | 0.37 | 118-138 |
| 26 | CUPVSiirt297 | 2 | 1.60 | 0.00 | 0.38 | 0.30 | 138-139 |
| 27 | CUPVSiirt298 | 4 | 2.29 | 0.50 | 0.56 | 0.52 | 116-152 |
| 28 | CUPVSiirt308 | 3 | 3.00 | 0.67 | 0.67 | 0.59 | 166-173 |
| 29 | CUPVSiirt316 | 2 | 1.60 | 0.00 | 0.38 | 0.30 | 262-264 |
| 30 | CUPVSiirt333 | 3 | 2.91 | 0.75 | 0.66 | 0.58 | 144-153 |
| 31 | CUPVSiirt340 | 2 | 2.00 | 0.50 | 0.50 | 0.38 | 129-157 |
| 32 | CUPVSiirt349 | 2 | 1.60 | 0.00 | 0.38 | 0.30 | 172-174 |
| 33 | CUPVSiirt357 | 3 | 2.46 | 0.50 | 0.59 | 0.51 | 188-202 |
| 34 | CUPVSiirt436 | 3 | 2.13 | 0.25 | 0.53 | 0.47 | 91-108 |
| 35 | CUPVSiirt446 | 3 | 2.46 | 1.00 | 0.59 | 0.51 | 221-242 |
| 36 | CUPVSiirt465 | 2 | 1.60 | 0.00 | 0.38 | 0.30 | 145-150 |
| 37 | CUPVSiirt476 | 3 | 1.68 | 0.50 | 0.41 | 0.37 | 149-165 |
| 38 | CUPVSiirt496 | 2 | 1.60 | 0.00 | 0.38 | 0.30 | 189-193 |
| 39 | CUPVSiirt501 | 3 | 2.91 | 0.25 | 0.66 | 0.58 | 146-158 |
| 40 | CUPVSiirt505 | 3 | 2.91 | 0.75 | 0.66 | 0.58 | 175-186 |
| 41 | CUPVSiirt509 | 3 | 2.00 | 0.33 | 0.50 | 0.45 | 164-180 |
| 42 | CUPVSiirt543 | 3 | 2.46 | 1.00 | 0.59 | 0.51 | 119-149 |
| 43 | CUPVSiirt565 | 3 | 2.13 | 0.75 | 0.53 | 0.47 | 134-152 |
| 44 | CUPVSiirt568y | 3 | 2.91 | 0.50 | 0.66 | 0.58 | 123-126 |
| 45 | CUPVSiirt569 | 2 | 1.28 | 0.25 | 0.22 | 0.19 | 96-102 |
| 46 | CUPVSiirt598 | 2 | 1.60 | 0.00 | 0.38 | 0.30 | 164-202 |
| 47 | CUPVSiirt600 | 2 | 1.88 | 0.75 | 0.47 | 0.36 | 227-231 |
| 48 | CUPVSiirt616 | 2 | 2.00 | 0.00 | 0.50 | 0.38 | 178-204 |
| 49 | CUPVSiirt617 | 2 | 1.80 | 0.00 | 0.44 | 0.35 | 281-290 |
| 50 | CUPVSiirt621 | 4 | 3.56 | 1.00 | 0.72 | 0.67 | 93-100 |
| 51 | CUPVSiirt625 | 4 | 3.20 | 0.75 | 0.69 | 0.63 | 165-175 |
| 52 | CUPVSiirt649 | 2 | 2.00 | 1.00 | 0.50 | 0.38 | 158-165 |
| 53 | CUPVSiirt660 | 3 | 2.91 | 0.75 | 0.66 | 0.58 | 131-135 |
| 54 | CUPVSiirt661 | 2 | 2.00 | 1.00 | 0.50 | 0.38 | 251-288 |
| 55 | CUPVSiirt689y | 2 | 2.00 | 1.00 | 0.50 | 0.38 | 306-314 |
| 56 | CUPVSiirt712 | 2 | 1.88 | 0.25 | 0.47 | 0.36 | 190-204 |
| 57 | CUPVSiirt715 | 3 | 2.13 | 0.50 | 0.53 | 0.47 | 139-143 |
| 58 | CUPVSiirt719 | 2 | 2.00 | 0.00 | 0.50 | 0.38 | 221-231 |
| 59 | CUPVSiirt724 | 2 | 2.00 | 1.00 | 0.50 | 0.38 | 132-135 |
| 60 | CUPVSiirt742 | 5 | 4.57 | 1.00 | 0.78 | 0.75 | 196-209 |
| 61 | CUPVSiirt764 | 2 | 2.00 | 1.00 | 0.50 | 0.38 | 164-176 |
| 62 | CUPVSiirt768 | 2 | 2.00 | 0.00 | 0.50 | 0.38 | 206220 |
| 63 | CUPVSiirt782 | 4 | 2.29 | 0.50 | 0.56 | 0.52 | 176-188 |
| 64 | CUPVSiirt788 | 2 | 2.00 | 0.00 | 0.50 | 0.38 | 214-216 |
| 65 | CUPVSiirt794 | 4 | 3.20 | 1.00 | 0.69 | 0.63 | 212-232 |
| 66 | CUPVSiirt796 | 3 | 2.13 | 0.75 | 0.53 | 0.47 | 104-116 |
| 67 | CUPVSiirt803 | 3 | 2.67 | 0.50 | 0.63 | 0.55 | 202-206 |
| 68 | CUPVSiirt818 | 3 | 1.68 | 0.50 | 0.41 | 0.37 | 171-177 |
| 69 | CUPVSiirt836 | 3 | 2.67 | 0.50 | 0.63 | 0.55 | 164-168 |
| 70 | CUPVSiirt838 | 5 | 4.00 | 1.00 | 0.75 | 0.71 | 149-163 |
| 71 | CUPVSiirt841 | 4 | 3.56 | 0.75 | 0.72 | 0.67 | 157-177 |
| 72 | CUPVSiirt855 | 2 | 2.00 | 0.00 | 0.50 | 0.38 | 248-250 |
| 73 | CUPVSiirt858 | 3 | 2.91 | 0.75 | 0.66 | 0.58 | 175-198 |
| 74 | CUPVSiirt875 | 3 | 2.67 | 0.50 | 0.63 | 0.55 | 163-169 |
| 75 | CUPVSiirt876 | 2 | 1.60 | 0.00 | 0.38 | 0.30 | 187-193 |
| 76 | CUPVSiirt883 | 2 | 2.00 | 0.00 | 0.50 | 0.38 | 174-185 |
| 77 | CUPVSiirt889 | 2 | 1.88 | 0.75 | 0.47 | 0.36 | 168-181 |
| 78 | CUPVSiirt891 | 2 | 2.00 | 0.50 | 0.50 | 0.38 | 142-148 |
| 79 | CUPVSiirt907 | 2 | 2.00 | 0.00 | 0.50 | 0.38 | 92-95 |
| 80 | CUPVSiirt924 | 2 | 2.00 | 0.00 | 0.50 | 0.38 | 84-87 |
| 81 | CUPVSiirt929 | 2 | 1.88 | 0.25 | 0.47 | 0.36 | 83-103 |
| 82 | CUPVSiirt949 | 2 | 2.00 | 1.00 | 0.50 | 0.38 | 167-179 |
| 83 | CUPVSiirt956 | 4 | 3.56 | 1.00 | 0.72 | 0.67 | 105-150 |
| 84 | CUPVSiirt961 | 2 | 1.88 | 0.75 | 0.47 | 0.36 | 190-192 |
| 85 | CUPVSiirt975 | 2 | 2.00 | 1.00 | 0.50 | 0.38 | 142-145 |
| 86 | CUPVSiirt989 | 3 | 1.68 | 0.50 | 0.41 | 0.37 | 152-164 |
| 87 | CUPVSiirt1003 | 2 | 2.00 | 0.00 | 0.50 | 0.38 | 71-75 |
| 88 | CUPVSiirt1017 | 2 | 1.88 | 0.75 | 0.47 | 0.36 | 205-245 |
| 89 | CUPVSiirt1021 | 3 | 2.46 | 0.25 | 0.59 | 0.51 | 123-131 |
| 90 | CUPVSiirt1041 | 3 | 2.13 | 0.75 | 0.53 | 0.47 | 103-107 |
| 91 | CUPVSiirt1043 | 2 | 1.60 | 0.50 | 0.38 | 0.30 | 89-127 |
| 92 | CUPVSiirt1047 | 2 | 2.00 | 1.00 | 0.50 | 0.38 | 125-133 |
| 93 | CUPVSiirt1055 | 2 | 2.00 | 0.33 | 0.50 | 0.38 | 157-163 |
| 94 | CUPVSiirt1057 | 2 | 1.60 | 0.00 | 0.38 | 0.30 | 237-252 |
| 95 | CUPVSiirt1071 | 2 | 2.00 | 1.00 | 0.50 | 0.38 | 123-133 |
| 96 | CUPVSiirt1092 | 3 | 3.00 | 0.67 | 0.67 | 0.59 | 151-165 |
| 97 | CUPVSiirt1095 | 2 | 1.80 | 0.00 | 0.44 | 0.35 | 240-242 |
| 98 | CUPVSiirt1116 | 2 | 1.88 | 0.75 | 0.47 | 0.36 | 151-162 |
| 99 | CUPVSiirt1117 | 2 | 2.00 | 0.50 | 0.50 | 0.38 | 150-158 |
| 100 | CUPVSiirt1120 | 2 | 2.00 | 0.00 | 0.50 | 0.38 | 197-198 |
| 101 | CUPVSiirt1127 | 3 | 2.91 | 0.75 | 0.66 | 0.58 | 118-134 |
| 102 | CUPVSiirt1140 | 2 | 2.00 | 1.00 | 0.50 | 0.38 | 157-162 |
| 103 | CUPVSiirt1145 | 2 | 1.60 | 0.50 | 0.38 | 0.30 | 154-164 |
| 104 | CUPVSiirt1163 | 2 | 1.38 | 0.33 | 0.28 | 0.24 | 140-152 |
| 105 | CUPVSiirt1171 | 3 | 2.13 | 0.75 | 0.53 | 0.47 | 230-255 |
| 106 | CUPVSiirt1182 | 3 | 2.13 | 0.50 | 0.53 | 0.47 | 155-179 |
| 107 | CUPVSiirt1183 | 2 | 1.88 | 0.25 | 0.47 | 0.36 | 235-237 |
| 108 | CUPVSiirt1191 | 2 | 2.00 | 0.50 | 0.50 | 0.38 | 162-164 |
| 109 | CUPVSiirt1202 | 3 | 2.46 | 0.50 | 0.59 | 0.51 | 178-186 |
| 110 | CUPVSiirt1214 | 2 | 1.60 | 0.00 | 0.38 | 0.30 | 180-182 |
| 111 | CUPVSiirt1224 | 2 | 1.60 | 0.00 | 0.38 | 0.30 | 257-273 |
| 112 | CUPVSiirt1238 | 4 | 3.20 | 1.00 | 0.69 | 0.63 | 233-255 |
| 113 | CUPVSiirt1243 | 4 | 3.60 | 0.33 | 0.72 | 0.67 | 138-150 |
| 114 | CUPVSiirt1250 | 3 | 2.67 | 1.00 | 0.63 | 0.55 | 176-192 |
| 115 | CUPVSiirt1267 | 2 | 2.00 | 1.00 | 0.50 | 0.38 | 133-149 |
| 116 | CUPVSiirt1273 | 2 | 2.00 | 1.00 | 0.50 | 0.38 | 140-154 |
| 117 | CUPVSiirt1278 | 3 | 2.67 | 1.00 | 0.63 | 0.55 | 176-181 |
| 118 | CUPVSiirt1322 | 4 | 3.56 | 0.25 | 0.72 | 0.67 | 218-226 |
| 119 | CUPVSiirt1326 | 2 | 1.28 | 0.25 | 0.22 | 0.19 | 186-211 |
| 120 | CUPVSiirt1330 | 5 | 3.20 | 0.75 | 0.69 | 0.65 | 157-185 |
| 121 | CUPVSiirt1345 | 3 | 2.46 | 0.25 | 0.59 | 0.51 | 173-191 |
| 122 | CUPVSiirt1353 | 2 | 1.28 | 0.25 | 0.22 | 0.19 | 169-197 |
| 123 | CUPVSiirt1372 | 4 | 2.29 | 0.50 | 0.56 | 0.52 | 112-129 |
| 124 | CUPVSiirt1378 | 2 | 1.88 | 0.25 | 0.47 | 0.36 | 84-97 |
| 125 | CUPVSiirt1388 | 3 | 2.13 | 0.75 | 0.53 | 0.47 | 177-183 |
| 126 | CUPVSiirt1400 | 3 | 2.67 | 1.00 | 0.63 | 0.55 | 166-172 |
| 127 | CUPVSiirt1402 | 3 | 2.91 | 0.25 | 0.66 | 0.58 | 174-181 |
| 128 | CUPVSiirt1405 | 2 | 1.38 | 0.33 | 0.28 | 0.24 | 185-219 |
| 129 | CUPVSiirt1406 | 2 | 1.60 | 0.00 | 0.38 | 0.30 | 196-197 |
| 130 | CUPVSiirt1413 | 2 | 2.00 | 0.75 | 0.50 | 0.51 | 172-190 |
| 131 | CUPVSiirt1418 | 2 | 2.00 | 0.00 | 0.50 | 0.38 | 144-153 |
| 132 | CUPVSiirt1431 | 2 | 1.88 | 0.25 | 0.47 | 0.36 | 209-213 |
| 133 | CUPVSiirt1438 | 2 | 2.00 | 0.00 | 0.50 | 0.38 | 277-280 |
| 134 | CUPVSiirt1442 | 2 | 1.88 | 0.25 | 0.47 | 0.36 | 122-128 |
| 135 | CUPVSiirt1457 | 3 | 2.46 | 0.50 | 0.59 | 0.51 | 189-199 |
| 136 | CUPVSiirt1477 | 3 | 2.13 | 0.75 | 0.53 | 0.47 | 115-119 |
| 137 | CUPVSiirt1517 | 3 | 2.46 | 0.50 | 0.59 | 0.51 | 213-221 |
| 138 | CUPVSiirt1547 | 2 | 1.60 | 0.50 | 0.38 | 0.30 | 120-126 |
| 139 | CUPVSiirt1564 | 3 | 2.13 | 0.50 | 0.53 | 0.47 | 208-216 |
| 140 | CUPVSiirt1567 | 3 | 1.68 | 0.50 | 0.41 | 0.37 | 191-215 |
| 141 | CUPVSiirt1611 | 2 | 1.88 | 0.25 | 0.47 | 0.36 | 193-205 |
| 142 | CUPVSiirt1626 | 2 | 1.28 | 0.25 | 0.22 | 0.19 | 124-125 |
| 143 | CUPVSiirt1639 | 2 | 2.00 | 0.00 | 0.50 | 0.38 | 170-176 |
| 144 | CUPVSiirt1640 | 3 | 2.67 | 0.50 | 0.63 | 0.55 | 144-174 |
| 145 | CUPVSiirt1652 | 3 | 2.46 | 0.50 | 0.59 | 0.51 | 172-178 |
| 146 | CUPVSiirt1655 | 3 | 2.13 | 0.75 | 0.53 | 0.47 | 158-192 |
| 147 | CUPVSiirt1658 | 4 | 3.20 | 0.75 | 0.69 | 0.63 | 136-167 |
| 148 | CUPVSiirt1667 | 3 | 2.91 | 0.25 | 0.66 | 0.58 | 164-170 |
| 149 | CUPVSiirt1705 | 2 | 1.60 | 0.50 | 0.38 | 0.30 | 241-251 |
| 150 | CUPVSiirt1714 | 2 | 1.38 | 0.33 | 0.28 | 0.24 | 196-207 |
| 151 | CUPVSiirt1740 | 2 | 1.28 | 0.25 | 0.22 | 0.19 | 162-168 |
| 152 | CUPVSiirt1742 | 2 | 1.28 | 0.25 | 0.22 | 0.19 | 179-207 |
| 153 | CUPVSiirt1749 | 4 | 3.56 | 0.75 | 0.72 | 0.67 | 145-154 |
| 154 | CUPVSiirt1759 | 4 | 3.00 | 1.00 | 0.67 | 0.62 | 151-159 |
| 155 | CUPVSiirt1768 | 2 | 2.00 | 0.00 | 0.50 | 0.38 | 123-128 |
| 156 | CUPVSiirt1784 | 3 | 1.68 | 0.50 | 0.41 | 0.37 | 193-205 |
| 157 | CUPVSiirt1797 | 3 | 2.91 | 0.25 | 0.66 | 0.58 | 155-159 |
|  | Total | 416 |  |  |  |  |  |
|  | Mean | 2.7 | 2.21 | 0.5 | 0.52 | 0.44 |  |
