# Supplementary material for: Genome survey of pistachio (Pistacia vera L.) by next generation sequencing: Development of novel SSR markers and genetic diversity in Pistacia species
Source: BMC Genomics. 2016 Dec 7;17:998. doi: 10.1186/s12864-016-3359-x (PMC5142174; doi:10.1186/s12864-016-3359-x)
Supplement: Additional file 5: — Genetic diversity measures in P. chinensis: allele ranges, number of alleles (Na), number of effective alleles (Ne), observed heterozygosity (Ho), expected heterozygosity (He), and PIC values of 142 polymorphic SSR loci. (DOCX 37 kb) [file 12864_2016_3359_MOESM5_ESM.docx]

**Additional file 5. Number of alleles (Na), Number of effective alleles (Ne), observed heterozygosity (Ho), expected heterozygosity (He), PIC values and allele range of 119 polymorphic SSR loci developed from *Pistacia chinensis.***

| **No** | **Loci** | **Na** | **Ne** | **Ho** | **He** | **PIC** | **Allele range**  **(bp)** |
| --- | --- | --- | --- | --- | --- | --- | --- |
| 1 | CUPVSiirt15 | 3 | 1.68 | 0.50 | 0.41 | 0.37 | 104-108 |
| 2 | CUPVSiirt22 | 2 | 1.88 | 0.25 | 0.47 | 0.36 | 180-181 |
| 3 | CUPVSiirt26 | 2 | 2.00 | 0.50 | 0.50 | 0.38 | 170-173 |
| 4 | CUPVSiirt37 | 2 | 1.28 | 0.25 | 0.22 | 0.19 | 155-167 |
| 5 | CUPVSiirt71 | 3 | 2.67 | 0.00 | 0.63 | 0.55 | 128-134 |
| 6 | CUPVSiirt95 | 2 | 1.28 | 0.25 | 0.22 | 0.19 | 196-216 |
| 7 | CUPVSiirt121 | 3 | 1.68 | 0.50 | 0.41 | 0.37 | 1118-124 |
| 8 | CUPVSiirt125 | 2 | 1.88 | 0.25 | 0.47 | 0.36 | 214-224 |
| 9 | CUPVSiirt129 | 4 | 2.91 | 0.75 | 0.66 | 0.60 | 140-169 |
| 10 | CUPVSiirt149 | 3 | 2.67 | 0.50 | 0.63 | 0.55 | 98-102 |
| 11 | CUPVSiirt151 | 2 | 1.88 | 0.25 | 0.47 | 0.36 | 158-172 |
| 12 | CUPVSiirt171 | 2 | 2.00 | 0.00 | 0.50 | 0.38 | 138-142 |
| 13 | CUPVSiirt186 | 6 | 4.57 | 0.75 | 0.78 | 0.75 | 160-167 |
| 14 | CUPVSiirt213 | 4 | 3.20 | 1.00 | 0.69 | 0.63 | 211-223 |
| 15 | CUPVSiirt230 | 3 | 2.13 | 0.75 | 0.53 | 0.47 | 177-187 |
| 16 | CUPVSiirt238 | 3 | 3.00 | 0.00 | 0.67 | 0.59 | 171-191 |
| 17 | CUPVSiirt242 | 3 | 2.46 | 1.00 | 0.59 | 0.51 | 129-138 |
| 18 | CUPVSiirt243 | 2 | 1.28 | 0.25 | 0.22 | 0.19 | 137-138 |
| 19 | CUPVSiirt256 | 3 | 2.57 | 0.33 | 0.61 | 0.54 | 173-183 |
| 20 | CUPVSiirt271 | 3 | 2.67 | 0.75 | 0.63 | 0.55 | 98-118 |
| 21 | CUPVSiirt284 | 4 | 2.91 | 0.50 | 0.66 | 0.60 | 229-251 |
| 22 | CUPVSiirt297 | 3 | 2.91 | 0.25 | 0.66 | 0.58 | 141-144 |
| 23 | CUPVSiirt308 | 2 | 2.00 | 0.00 | 0.50 | 0.38 | 169-170 |
| 24 | CUPVSiirt312 | 5 | 4.57 | 0.50 | 0.78 | 0.75 | 154-172 |
| 25 | CUPVSiirt333 | 5 | 3.20 | 1.00 | 0.69 | 0.65 | 142-150 |
| 26 | CUPVSiirt349 | 3 | 2.57 | 1.00 | 0.61 | 0.54 | 164-170 |
| 27 | CUPVSiirt357 | 2 | 1.60 | 0.50 | 0.38 | 0.30 | 181-185 |
| 28 | CUPVSiirt436 | 4 | 3.20 | 0.75 | 0.69 | 0.63 | 91-101 |
| 29 | CUPVSiirt472 | 5 | 4.00 | 1.00 | 0.75 | 0.71 | 181-203 |
| 30 | CUPVSiirt476 | 2 | 2.00 | 0.00 | 0.50 | 0.38 | 145-149 |
| 31 | CUPVSiirt496 | 4 | 3.60 | 0.67 | 0.72 | 0.67 | 203-239 |
| 32 | CUPVSiirt501 | 2 | 2.00 | 1.00 | 0.50 | 0.38 | 148-158 |
| 33 | CUPVSiirt505 | 3 | 2.13 | 0.25 | 0.53 | 0.47 | 154-170 |
| 34 | CUPVSiirt509 | 2 | 1.60 | 0.50 | 0.38 | 0.30 | 168-175 |
| 35 | CUPVSiirt543 | 3 | 2.67 | 0.50 | 0.63 | 0.55 | 111-125 |
| 36 | CUPVSiirt565 | 5 | 4.57 | 0.50 | 0.78 | 0.75 | 147-174 |
| 37 | CUPVSiirt568x | 2 | 2.00 | 1.00 | 0.50 | 0.38 | 109-117 |
| 38 | CUPVSiirt625 | 3 | 2.67 | 0.00 | 0.63 | 0.55 | 183-191 |
| 39 | CUPVSiirt649 | 3 | 2.91 | 1.00 | 0.66 | 0.58 | 165-177 |
| 40 | CUPVSiirt660 | 3 | 2.00 | 0.33 | 0.50 | 0.45 | 131-142 |
| 41 | CUPVSiirt712 | 2 | 1.80 | 0.00 | 0.44 | 0.35 | 204-210 |
| 42 | CUPVSiirt719 | 2 | 1.60 | 0.50 | 0.38 | 0.3 | 209-212 |
| 43 | CUPVSiirt724 | 2 | 1.28 | 0.25 | 0.22 | 0.19 | 1322-135 |
| 44 | CUPVSiirt742 | 2 | 1.28 | 0.25 | 0.22 | 0.19 | 205-209 |
| 45 | CUPVSiirt768 | 3 | 1.68 | 0.25 | 0.41 | 0.37 | 208-220 |
| 46 | CUPVSiirt782 | 2 | 1.60 | 0.50 | 0.38 | 0.3 | 176-188 |
| 47 | CUPVSiirt788 | 5 | 3.20 | 0.50 | 0.69 | 0.65 | 205-262 |
| 48 | CUPVSiirt796 | 4 | 2.29 | 0.75 | 0.56 | 0.52 | 96-106 |
| 49 | CUPVSiirt818 | 4 | 3.56 | 1.00 | 0.72 | 0.67 | 173-187 |
| 50 | CUPVSiirt836 | 7 | 6.40 | 1.00 | 0.84 | 0.82 | 152-174 |
| 51 | CUPVSiirt838 | 3 | 1.68 | 0.25 | 0.41 | 0.37 | 142-153 |
| 52 | CUPVSiirt855 | 2 | 1.28 | 0.25 | 0.22 | 0.19 | 250-278 |
| 53 | CUPVSiirt858 | 2 | 1.88 | 0.75 | 0.47 | 0.36 | 178-180 |
| 54 | CUPVSiirt875 | 4 | 4.00 | 0.00 | 0.75 | 0.70 | 161-185 |
| 55 | CUPVSiirt876 | 5 | 4.57 | 0.75 | 0.78 | 0.75 | 180-189 |
| 56 | CUPVSiirt883 | 2 | 1.80 | 0.67 | 0.44 | 0.35 | 179-181 |
| 57 | CUPVSiirt929 | 3 | 2.13 | 0.25 | 0.53 | 0.47 | 88-95 |
| 58 | CUPVSiirt961 | 2 | 2.00 | 1.00 | 0.50 | 0.38 | 177-179 |
| 59 | CUPVSiirt986 | 2 | 1.88 | 0.25 | 0.47 | 0.36 | 156-157 |
| 60 | CUPVSiirt989 | 3 | 2.91 | 0.25 | 0.66 | 0.58 | 146-162 |
| 61 | CUPVSiirt1003 | 2 | 1.88 | 0.75 | 0.47 | 0.36 | 73-96 |
| 62 | CUPVSiirt1021 | 2 | 1.60 | 0.00 | 0.38 | 0.30 | 127-143 |
| 63 | CUPVSiirt1047 | 2 | 1.38 | 0.33 | 0.28 | 0.24 | 125-131 |
| 64 | CUPVSiirt1053 | 2 | 1.38 | 0.33 | 0.28 | 0.24 | 159-166 |
| 65 | CUPVSiirt1062 | 4 | 2.29 | 0.75 | 0.56 | 0.52 | 144-172 |
| 66 | CUPVSiirt1071 | 2 | 2.00 | 1.00 | 0.50 | 0.38 | 131-143 |
| 67 | CUPVSiirt1116 | 3 | 1.68 | 0.50 | 0.41 | 0.37 | 154-170 |
| 68 | CUPVSiirt1117 | 2 | 2.00 | 0.00 | 0.50 | 0.38 | 148-149 |
| 69 | CUPVSiirt1120 | 2 | 1.60 | 0.00 | 0.38 | 0.30 | 198-201 |
| 70 | CUPVSiirt1122 | 2 | 2.00 | 1.00 | 0.50 | 0.38 | 121-210 |
| 71 | CUPVSiirt1140 | 4 | 4.00 | 1.00 | 0.75 | 0.70 | 156-165 |
| 72 | CUPVSiirt1145 | 3 | 2.67 | 0.75 | 0.63 | 0.55 | 154-166 |
| 73 | CUPVSiirt1153 | 2 | 2.00 | 1.00 | 0.50 | 0.38 | 176-194 |
| 74 | CUPVSiirt1171 | 5 | 4.00 | 0.75 | 0.75 | 0.71 | 229-261 |
| 75 | CUPVSiirt1182 | 2 | 1.28 | 0.25 | 0.22 | 0.19 | 161-173 |
| 76 | CUPVSiirt1202 | 5 | 4.00 | 1.00 | 0.75 | 0.71 | 190-212 |
| 77 | CUPVSiirt1214 | 2 | 2.00 | 0.00 | 0.50 | 0.38 | 175-177 |
| 78 | CUPVSiirt1224 | 4 | 2.91 | 0.75 | 0.66 | 0.60 | 265-292 |
| 79 | CUPVSiirt1238 | 4 | 2.91 | 0.50 | 0.66 | 0.60 | 239-258 |
| 80 | CUPVSiirt1243 | 2 | 2.00 | 0.00 | 0.50 | 0.38 | 140-142 |
| 81 | CUPVSiirt1250 | 2 | 1.60 | 0.00 | 0.38 | 0.30 | 176-177 |
| 82 | CUPVSiirt1260 | 4 | 2.91 | 0.75 | 0.66 | 0.60 | 154-184 |
| 83 | CUPVSiirt1271 | 2 | 1.28 | 0.25 | 0.22 | 0.19 | 224-226 |
| 84 | CUPVSiirt1273 | 2 | 1.28 | 0.25 | 0.22 | 0.19 | 139-140 |
| 85 | CUPVSiirt1278 | 5 | 3.20 | 0.75 | 0.69 | 0.65 | 177-204 |
| 86 | CUPVSiirt1326 | 3 | 2.13 | 0.25 | 0.53 | 0.47 | 186-212 |
| 87 | CUPVSiirt1330 | 6 | 5.33 | 1.00 | 0.81 | 0.79 | 165-201 |
| 88 | CUPVSiirt1345 | 4 | 3.60 | 0.67 | 0.72 | 0.67 | 156-191 |
| 89 | CUPVSiirt1372 | 2 | 2.00 | 0.00 | 0.50 | 0.38 | 123-130 |
| 90 | CUPVSiirt1378 | 3 | 2.13 | 0.50 | 0.53 | 0.47 | 84-99 |
| 91 | CUPVSiirt1400 | 6 | 5.33 | 1.00 | 0.81 | 0.79 | 155-178 |
| 92 | CUPVSiirt1402 | 2 | 1.60 | 0.50 | 0.38 | 0.30 | 172-181 |
| 93 | CUPVSiirt1405 | 3 | 2.91 | 0.25 | 0.66 | 0.58 | 201-216 |
| 94 | CUPVSiirt1406 | 3 | 1.68 | 0.50 | 0.41 | 0.37 | 188-198 |
| 95 | CUPVSiirt1431 | 3 | 2.46 | 0.75 | 0.59 | 0.51 | 200-211 |
| 96 | CUPVSiirt1438 | 2 | 1.60 | 0.00 | 0.38 | 0.30 | 283-286 |
| 97 | CUPVSiirt1442 | 4 | 4.00 | 0.00 | 0.75 | 0.70 | 114-124 |
| 98 | CUPVSiirt1477 | 4 | 3.56 | 1.00 | 0.72 | 0.67 | 112-126 |
| 99 | CUPVSiirt1478 | 3 | 1.68 | 0.50 | 0.41 | 0.37 | 92-96 |
| 100 | CUPVSiirt1564 | 5 | 4.50 | 0.67 | 0.78 | 0.74 | 208-222 |
| 101 | CUPVSiirt1567 | 4 | 4.00 | 0.00 | 0.75 | 0.70 | 181-193 |
| 102 | CUPVSiirt1611 | 3 | 2.13 | 0.25 | 0.53 | 0.47 | 193-203 |
| 103 | CUPVSiirt1626 | 3 | 2.67 | 0.00 | 0.63 | 0.55 | 125-130 |
| 104 | CUPVSiirt1628 | 3 | 2.13 | 0.75 | 0.53 | 0.47 | 127-146 |
| 105 | CUPVSiirt1639 | 2 | 1.38 | 0.33 | 0.28 | 0.24 | 168-170 |
| 106 | CUPVSiirt1652 | 4 | 3.56 | 1.00 | 0.72 | 0.67 | 159-182 |
| 107 | CUPVSiirt1655 | 3 | 1.68 | 0.50 | 0.41 | 0.37 | 158-163 |
| 108 | CUPVSiirt1658 | 5 | 4.57 | 0.75 | 0.78 | 0.75 | 134-173 |
| 109 | CUPVSiirt1667 | 3 | 2.00 | 0.67 | 0.50 | 0.45 | 164-170 |
| 110 | CUPVSiirt1705 | 2 | 1.80 | 0.00 | 0.44 | 0.35 | 241-251 |
| 111 | CUPVSiirt1714 | 2 | 1.60 | 0.50 | 0.38 | 0.30 | 202-208 |
| 112 | CUPVSiirt1740 | 4 | 3.56 | 1.00 | 0.72 | 0.67 | 168-192 |
| 113 | CUPVSiirt1742 | 3 | 2.67 | 0.00 | 0.63 | 0.55 | 161-191 |
| 114 | CUPVSiirt1749 | 3 | 2.46 | 0.25 | 0.59 | 0.51 | 143-158 |
| 115 | CUPVSiirt1759 | 3 | 2.91 | 0.25 | 0.66 | 0.58 | 141-146 |
| 116 | CUPVSiirt1764 | 3 | 2.46 | 0.25 | 0.59 | 0.51 | 157-162 |
| 117 | CUPVSiirt1768 | 4 | 3.60 | 1.00 | 0.72 | 0.67 | 108-119 |
| 118 | CUPVSiirt1784 | 2 | 1.80 | 0.00 | 0.44 | 0.35 | 177-193 |
| 119 | CUPVSiirt1788 | 2 | 2.00 | 0.33 | 0.50 | 0.38 | 183-185 |
|  | Total | 365 |  |  |  |  |  |
|  | Mean | 3.1 | 2.51 | 0.48 | 0.54 | 0.48 |  |
