# Supplementary material for: Genome survey of pistachio (Pistacia vera L.) by next generation sequencing: Development of novel SSR markers and genetic diversity in Pistacia species
Source: BMC Genomics. 2016 Dec 7;17:998. doi: 10.1186/s12864-016-3359-x (PMC5142174; doi:10.1186/s12864-016-3359-x)
Supplement: Additional file 6: — Genetic diversity measures in P. terebinthus: allele ranges, number of alleles (Na), number of effective alleles (Ne), observed heterozygosity (Ho), expected heterozygosity (He), and PIC values of 119 polymorphic SSR loci. (DOCX 38 kb) [file 12864_2016_3359_MOESM6_ESM.docx]

**Additional file 6. Number of alleles (Na), Number of effective alleles (Ne), observed heterozygosity (Ho), expected heterozygosity (He), PIC values and allele range of 142 polymorphic SSR loci developed from *Pistacia terebinthus.***

| **No** | **Loci** | **Na** | **Ne** | **Ho** | **He** | **PIC** | **Allele range**  **(bp)** |
| --- | --- | --- | --- | --- | --- | --- | --- |
| 1 | CUPVSiirt15 | 3 | 2.67 | 0.00 | 0.63 | 0.55 | 96-104 |
| 2 | CUPVSiirt22 | 2 | 1.88 | 0.75 | 0.47 | 0.36 | 160-181 |
| 3 | CUPVSiirt26 | 2 | 1.28 | 0.25 | 0.22 | 0.19 | 170-179 |
| 4 | CUPVSiirt37 | 2 | 2.00 | 0.00 | 0.50 | 0.38 | 148-164 |
| 5 | CUPVSiirt50 | 5 | 4.57 | 0.50 | 0.78 | 0.75 | 161-184 |
| 6 | CUPVSiirt71 | 2 | 1.80 | 0.00 | 0.44 | 0.35 | 132-134 |
| 7 | CUPVSiirt76 | 2 | 1.28 | 0.25 | 0.22 | 0.19 | 172-174 |
| 8 | CUPVSiirt86 | 4 | 3.56 | 0.75 | 0.72 | 0.67 | 126-151 |
| 9 | CUPVSiirt95 | 4 | 2.91 | 0.25 | 0.66 | 0.6 | 191-235 |
| 10 | CUPVSiirt121 | 3 | 2.46 | 0.25 | 0.59 | 0.51 | 121-127 |
| 11 | CUPVSiirt125 | 5 | 4.57 | 0.75 | 0.78 | 0.75 | 175-203 |
| 12 | CUPVSiirt129 | 4 | 2.29 | 0.50 | 0.56 | 0.52 | 143-172 |
| 13 | CUPVSiirt131 | 3 | 2.46 | 0.50 | 0.59 | 0.51 | 156-170 |
| 14 | CUPVSiirt149 | 3 | 1.68 | 0.50 | 0.41 | 0.37 | 103-106 |
| 15 | CUPVSiirt151 | 3 | 1.68 | 0.25 | 0.41 | 0.37 | 150-168 |
| 16 | CUPVSiirt186 | 2 | 1.28 | 0.25 | 0.22 | 0.19 | 160-170 |
| 17 | CUPVSiirt213 | 3 | 1.68 | 0.50 | 0.41 | 0.37 | 297-211 |
| 18 | CUPVSiirt230 | 3 | 2.91 | 0.50 | 0.66 | 0.58 | 177-186 |
| 19 | CUPVSiirt242 | 2 | 1.88 | 0.25 | 0.47 | 0.36 | 134-136 |
| 20 | CUPVSiirt243 | 6 | 5.33 | 0.75 | 0.81 | 0.79 | 144-166 |
| 21 | CUPVSiirt256 | 2 | 2.00 | 0.00 | 0.50 | 0.38 | 177-183 |
| 22 | CUPVSiirt259 | 3 | 2.46 | 0.50 | 0.59 | 0.51 | 194-198 |
| 23 | CUPVSiirt271 | 4 | 2.91 | 0.75 | 0.66 | 0.6 | 96-110 |
| 24 | CUPVSiirt284 | 2 | 2.00 | 0.00 | 0.50 | 0.38 | 239-248 |
| 25 | CUPVSiirt294 | 2 | 1.28 | 0.25 | 0.22 | 0.19 | 116-118 |
| 26 | CUPVSiirt297 | 3 | 2.13 | 0.25 | 0.53 | 0.47 | 139-141 |
| 27 | CUPVSiirt298 | 5 | 4.00 | 1.00 | 0.75 | 0.71 | 106-198 |
| 28 | CUPVSiirt308 | 3 | 2.67 | 0.50 | 0.63 | 0.55 | 166-173 |
| 29 | CUPVSiirt312 | 4 | 3.56 | 0.75 | 0.72 | 0.67 | 154-168 |
| 30 | CUPVSiirt349 | 3 | 2.67 | 1.00 | 0.63 | 0.55 | 168-179 |
| 31 | CUPVSiirt357 | 3 | 1.68 | 0.50 | 0.41 | 0.37 | 185-192 |
| 32 | CUPVSiirt436 | 4 | 3.56 | 0.25 | 0.72 | 0.67 | 92-118 |
| 33 | CUPVSiirt465 | 5 | 4.00 | 0.75 | 0.75 | 0.71 | 143-177 |
| 34 | CUPVSiirt472 | 3 | 2.13 | 0.25 | 0.53 | 0.47 | 206-237 |
| 35 | CUPVSiirt476 | 2 | 1.28 | 0.25 | 0.22 | 0.19 | 146-149 |
| 36 | CUPVSiirt479 | 2 | 1.38 | 0.33 | 0.28 | 0.24 | 165-187 |
| 37 | CUPVSiirt496 | 2 | 2.00 | 0.00 | 0.50 | 0.38 | 158-163 |
| 38 | CUPVSiirt501 | 3 | 2.67 | 0.50 | 0.63 | 0.55 | 153-171 |
| 39 | CUPVSiirt505 | 4 | 3.56 | 0.75 | 0.72 | 0.67 | 154-186 |
| 40 | CUPVSiirt509 | 2 | 1.28 | 0.25 | 0.22 | 0.19 | 175-180 |
| 41 | CUPVSiirt543 | 3 | 2.13 | 0.50 | 0.53 | 0.47 | 119-140 |
| 42 | CUPVSiirt565 | 6 | 5.33 | 0.50 | 0.81 | 0.79 | 138-154 |
| 43 | CUPVSiirt568y | 3 | 1.68 | 0.50 | 0.41 | 0.37 | 121-126 |
| 44 | CUPVSiirt569 | 2 | 1.28 | 0.25 | 0.22 | 0.19 | 102-109 |
| 45 | CUPVSiirt598 | 5 | 4.50 | 1.00 | 0.78 | 0.74 | 178-204 |
| 46 | CUPVSiirt600 | 2 | 1.60 | 0.00 | 0.38 | 0.30 | 225-227 |
| 47 | CUPVSiirt616 | 2 | 2.00 | 0.00 | 0.50 | 0.38 | 138-140 |
| 48 | CUPVSiirt621 | 2 | 1.60 | 0.00 | 0.38 | 0.30 | 102-103 |
| 49 | CUPVSiirt625 | 5 | 4.57 | 0.25 | 0.78 | 0.75 | 175-185 |
| 50 | CUPVSiirt649 | 2 | 1.28 | 0.25 | 0.22 | 0.19 | 170-177 |
| 51 | CUPVSiirt660 | 4 | 3.60 | 1.00 | 0.72 | 0.67 | 129-146 |
| 52 | CUPVSiirt712 | 2 | 1.28 | 0.25 | 0.22 | 0.19 | 186-190 |
| 53 | CUPVSiirt715 | 3 | 2.13 | 0.50 | 0.53 | 0.47 | 139-158 |
| 54 | CUPVSiirt719 | 3 | 2.57 | 1.00 | 0.61 | 0.54 | 212-234 |
| 55 | CUPVSiirt742 | 6 | 5.33 | 1.00 | 0.81 | 0.79 | 198-208 |
| 56 | CUPVSiirt768 | 3 | 2.13 | 0.75 | 0.53 | 0.47 | 210-220 |
| 57 | CUPVSiirt782 | 2 | 1.60 | 0.50 | 0.38 | 0.30 | 176-188 |
| 58 | CUPVSiirt788 | 6 | 5.33 | 1.00 | 0.81 | 0.79 | 210-248 |
| 59 | CUPVSiirt794 | 5 | 4.00 | 0.75 | 0.75 | 0.71 | 216-230 |
| 60 | CUPVSiirt796 | 6 | 4.57 | 1.00 | 0.78 | 0.75 | 92-115 |
| 61 | CUPVSiirt803 | 2 | 1.88 | 0.75 | 0.47 | 0.36 | 258-266 |
| 62 | CUPVSiirt818 | 3 | 2.13 | 0.50 | 0.53 | 0.47 | 177-183 |
| 63 | CUPVSiirt836 | 5 | 3.20 | 0.75 | 0.69 | 0.65 | 148-162 |
| 64 | CUPVSiirt838 | 6 | 5.33 | 0.75 | 0.81 | 0.79 | 149-170 |
| 65 | CUPVSiirt841 | 5 | 4.50 | 0.67 | 0.78 | 0.74 | 146-171 |
| 66 | CUPVSiirt855 | 2 | 1.60 | 0.50 | 0.38 | 0.30 | 248-252 |
| 67 | CUPVSiirt858 | 2 | 1.28 | 0.25 | 0.22 | 0.19 | 175-177 |
| 68 | CUPVSiirt875 | 5 | 4.00 | 0.50 | 0.75 | 0.71 | 165-190 |
| 69 | CUPVSiirt876 | 6 | 5.33 | 1.00 | 0.81 | 0.79 | 191-221 |
| 70 | CUPVSiirt883 | 5 | 4.00 | 0.50 | 0.75 | 0.71 | 179-208 |
| 71 | CUPVSiirt889 | 2 | 1.60 | 0.00 | 0.38 | 0.30 | 177-181 |
| 72 | CUPVSiirt891 | 3 | 1.68 | 0.50 | 0.41 | 0.37 | 142-168 |
| 73 | CUPVSiirt929 | 2 | 1.88 | 0.75 | 0.47 | 0.36 | 83-85 |
| 74 | CUPVSiirt949 | 2 | 1.28 | 0.25 | 0.22 | 0.19 | 167-171 |
| 75 | CUPVSiirt951 | 3 | 2.67 | 0.00 | 0.63 | 0.55 | 154-184 |
| 76 | CUPVSiirt956 | 2 | 2.00 | 0.00 | 0.50 | 0.38 | 131-141 |
| 77 | CUPVSiirt975 | 2 | 1.60 | 0.00 | 0.38 | 0.30 | 139-140 |
| 78 | CUPVSiirt986 | 4 | 2.29 | 0.75 | 0.56 | 0.52 | 157-165 |
| 79 | CUPVSiirt989 | 5 | 4.00 | 0.75 | 0.75 | 0.71 | 140-168 |
| 80 | CUPVSiirt1003 | 4 | 3.60 | 0.33 | 0.72 | 0.67 | 73-97 |
| 81 | CUPVSiirt1008 | 2 | 1.60 | 0.00 | 0.38 | 0.30 | 165-168 |
| 82 | CUPVSiirt1017 | 7 | 6.40 | 0.75 | 0.84 | 0.82 | 214-239 |
| 83 | CUPVSiirt1043 | 2 | 1.28 | 0.25 | 0.22 | 0.19 | 98-101 |
| 84 | CUPVSiirt1047 | 3 | 2.91 | 0.75 | 0.66 | 0.58 | 127-155 |
| 85 | CUPVSiirt1053 | 2 | 1.60 | 0.50 | 0.38 | 0.30 | 166-172 |
| 86 | CUPVSiirt1055 | 2 | 2.00 | 0.33 | 0.50 | 0.38 | 151-163 |
| 87 | CUPVSiirt1057 | 6 | 5.33 | 0.75 | 0.81 | 0.79 | 226-252 |
| 88 | CUPVSiirt1062 | 3 | 2.91 | 0.75 | 0.66 | 0.58 | 144-159 |
| 89 | CUPVSiirt1071 | 4 | 2.91 | 0.75 | 0.66 | 0.60 | 140-147 |
| 90 | CUPVSiirt1116 | 6 | 5.33 | 1.00 | 0.81 | 0.79 | 158-188 |
| 91 | CUPVSiirt1117 | 2 | 1.60 | 0.50 | 0.38 | 0.30 | 149-150 |
| 92 | CUPVSiirt1120 | 2 | 1.28 | 0.25 | 0.22 | 0.19 | 197-208 |
| 93 | CUPVSiirt1127 | 5 | 4.00 | 1.00 | 0.75 | 0.71 | 120-142 |
| 94 | CUPVSiirt1145 | 2 | 1.28 | 0.25 | 0.22 | 0.19 | 162-168 |
| 95 | CUPVSiirt1153 | 2 | 1.28 | 0.25 | 0.22 | 0.19 | 182-194 |
| 96 | CUPVSiirt1171 | 6 | 5.33 | 0.75 | 0.81 | 0.79 | 229-249 |
| 97 | CUPVSiirt1182 | 3 | 2.67 | 0.00 | 0.63 | 0.55 | 155-173 |
| 98 | CUPVSiirt1183 | 2 | 1.60 | 0.00 | 0.38 | 0.30 | 240-243 |
| 99 | CUPVSiirt1202 | 5 | 4.57 | 1.00 | 0.78 | 0.75 | 180-202 |
| 100 | CUPVSiirt1224 | 3 | 2.67 | 0.00 | 0.63 | 0.55 | 265-275 |
| 101 | CUPVSiirt1238 | 3 | 2.46 | 0.25 | 0.59 | 0.51 | 238-242 |
| 102 | CUPVSiirt1243 | 5 | 4.57 | 0.25 | 0.78 | 0.75 | 134-164 |
| 103 | CUPVSiirt1260 | 3 | 2.46 | 0.75 | 0.59 | 0.51 | 160-175 |
| 104 | CUPVSiirt1271 | 2 | 1.28 | 0.25 | 0.22 | 0.19 | 231-234 |
| 105 | CUPVSiirt1273 | 2 | 1.60 | 0.50 | 0.38 | 0.30 | 135-147 |
| 106 | CUPVSiirt1278 | 3 | 2.13 | 0.25 | 0.53 | 0.47 | 177-194 |
| 107 | CUPVSiirt1322 | 3 | 2.67 | 0.00 | 0.63 | 0.55 | 220-222 |
| 108 | CUPVSiirt1326 | 7 | 6.40 | 1.00 | 0.84 | 0.82 | 187-208 |
| 109 | CUPVSiirt1330 | 2 | 1.80 | 0.00 | 0.44 | 0.35 | 154-173 |
| 110 | CUPVSiirt1331 | 2 | 1.28 | 0.25 | 0.22 | 0.19 | 97-98 |
| 111 | CUPVSiirt1353 | 3 | 2.91 | 0.25 | 0.66 | 0.58 | 183-187 |
| 112 | CUPVSiirt1372 | 4 | 3.00 | 0.67 | 0.67 | 0.62 | 115-130 |
| 113 | CUPVSiirt1378 | 6 | 5.33 | 0.75 | 0.81 | 0.79 | 87-119 |
| 114 | CUPVSiirt1394 | 2 | 1.60 | 0.50 | 0.38 | 0.30 | 234-236 |
| 115 | CUPVSiirt1399 | 2 | 1.28 | 0.25 | 0.22 | 0.19 | 204-210 |
| 116 | CUPVSiirt1400 | 6 | 5.33 | 1.00 | 0.81 | 0.79 | 164-184 |
| 117 | CUPVSiirt1402 | 2 | 1.28 | 0.25 | 0.22 | 0.19 | 178-181 |
| 118 | CUPVSiirt1405 | 7 | 6.40 | 0.75 | 0.84 | 0.82 | 183-219 |
| 119 | CUPVSiirt1406 | 7 | 6.40 | 0.75 | 0.84 | 0.82 | 188-208 |
| 120 | CUPVSiirt1413 | 4 | 2.91 | 1.00 | 0.66 | 0.60 | 182-202 |
| 121 | CUPVSiirt1417 | 3 | 3.00 | 0.00 | 0.67 | 0.59 | 134-150 |
| 122 | CUPVSiirt1418 | 4 | 2.91 | 0.75 | 0.66 | 0.60 | 131-138 |
| 123 | CUPVSiirt1431 | 2 | 1.28 | 0.25 | 0.22 | 0.19 | 198-200 |
| 124 | CUPVSiirt1442 | 5 | 3.20 | 0.75 | 0.69 | 0.65 | 120-138 |
| 125 | CUPVSiirt1477 | 3 | 2.13 | 0.75 | 0.53 | 0.47 | 116-118 |
| 126 | CUPVSiirt1564 | 4 | 2.91 | 0.50 | 0.66 | 0.60 | 188-208 |
| 127 | CUPVSiirt1567 | 4 | 3.56 | 0.25 | 0.72 | 0.67 | 189-205 |
| 128 | CUPVSiirt1611 | 2 | 2.00 | 0.00 | 0.50 | 0.38 | 198-203 |
| 129 | CUPVSiirt1626 | 3 | 2.67 | 0.00 | 0.63 | 0.55 | 130-134 |
| 130 | CUPVSiirt1628 | 4 | 2.91 | 0.75 | 0.66 | 0.60 | 127-155 |
| 131 | CUPVSiirt1652 | 3 | 2.67 | 0.50 | 0.63 | 0.55 | 159-176 |
| 132 | CUPVSiirt1655 | 2 | 2.00 | 0.00 | 0.50 | 0.38 | 156-158 |
| 133 | CUPVSiirt1658 | 4 | 2.91 | 0.75 | 0.66 | 0.60 | 199-209 |
| 134 | CUPVSiirt1667 | 4 | 3.60 | 0.33 | 0.72 | 0.67 | 168-174 |
| 135 | CUPVSiirt1688 | 3 | 2.46 | 0.50 | 0.59 | 0.51 | 160-174 |
| 136 | CUPVSiirt1714 | 2 | 2.00 | 0.00 | 0.5 | 0.38 | 202-208 |
| 137 | CUPVSiirt1742 | 4 | 4.00 | 0.00 | 0.75 | 0.70 | 167-203 |
| 138 | CUPVSiirt1749 | 4 | 3.60 | 0.67 | 0.72 | 0.67 | 143-161 |
| 139 | CUPVSiirt1759 | 4 | 3.56 | 1.00 | 0.72 | 0.67 | 137-146 |
| 140 | CUPVSiirt1764 | 5 | 4.57 | 0.75 | 0.78 | 0.75 | 161-172 |
| 141 | CUPVSiirt1768 | 4 | 2.91 | 1.00 | 0.66 | 0.60 | 108-123 |
| 142 | CUPVSiirt1788 | 2 | 1.28 | 0.25 | 0.22 | 0.19 | 183-185 |
|  | Total | 485 |  |  |  |  |  |
|  | Mean | 3.4 | 2.8 | 0.47 | 0.56 | 0.5 |  |
