# Supplementary material for: Genome survey of pistachio (Pistacia vera L.) by next generation sequencing: Development of novel SSR markers and genetic diversity in Pistacia species
Source: BMC Genomics. 2016 Dec 7;17:998. doi: 10.1186/s12864-016-3359-x (PMC5142174; doi:10.1186/s12864-016-3359-x)
Supplement: Additional file 7: — Genetic diversity measures in P. lentiscus: allele ranges, number of alleles (Na), number of effective alleles (Ne), observed heterozygosity (Ho), expected heterozygosity (He), and PIC values of 83 polymorphic SSR loci. (DOCX 34 kb) [file 12864_2016_3359_MOESM7_ESM.docx]

**Additional file 7. Number of alleles (Na), Number of effective alleles (Ne), observed heterozygosity (Ho), expected heterozygosity (He), PIC values and allele range of 83 polymorphic SSR primer loci from *Pistacia lentiscus.***

| **No** | **Loci** | **Na** | **Ne** | **Ho** | **He** | **PIC** | **Allele range**  **(bp)** |
| --- | --- | --- | --- | --- | --- | --- | --- |
| 1 | CUPVSiirt26 | 2 | 2.00 | 1.00 | 0.5 | 0.38 | 179-188 |
| 2 | CUPVSiirt37 | 3 | 2.46 | 0.75 | 0.59 | 0.51 | 148-163 |
| 3 | CUPVSiirt86 | 4 | 2.29 | 0.75 | 0.56 | 0.52 | 112-124 |
| 4 | CUPVSiirt95 | 2 | 2.00 | 1.00 | 0.50 | 0.38 | 217-219 |
| 5 | CUPVSiirt129 | 2 | 1.60 | 0.50 | 0.38 | 0.30 | 127-137 |
| 6 | CUPVSiirt171 | 2 | 1.80 | 0.67 | 0.44 | 0.35 | 138-149 |
| 7 | CUPVSiirt230 | 3 | 2.46 | 0.50 | 0.59 | 0.51 | 185-193 |
| 8 | CUPVSiirt242 | 4 | 2.91 | 0.75 | 0.66 | 0.60 | 134-147 |
| 9 | CUPVSiirt256 | 2 | 1.60 | 0.50 | 0.38 | 0.30 | 173-187 |
| 10 | CUPVSiirt284 | 2 | 1.88 | 0.25 | 0.47 | 0.36 | 252-256 |
| 11 | CUPVSiirt294 | 3 | 2.91 | 0.75 | 0.66 | 0.58 | 114-120 |
| 12 | CUPVSiirt297 | 2 | 1.60 | 0.00 | 0.38 | 0.30 | 139-140 |
| 13 | CUPVSiirt312 | 3 | 3.00 | 0.00 | 0.67 | 0.59 | 158-166 |
| 14 | CUPVSiirt333 | 3 | 2.13 | 0.75 | 0.53 | 0.47 | 151-159 |
| 15 | CUPVSiirt340 | 3 | 2.13 | 0.75 | 0.53 | 0.47 | 131-135 |
| 16 | CUPVSiirt349 | 3 | 2.57 | 1.00 | 0.61 | 0.54 | 168-178 |
| 17 | CUPVSiirt436 | 2 | 2.00 | 1.00 | 0.50 | 0.38 | 100-101 |
| 18 | CUPVSiirt446 | 2 | 1.28 | 0.25 | 0.22 | 0.19 | 236-242 |
| 19 | CUPVSiirt465 | 2 | 1.60 | 0.50 | 0.38 | 0.30 | 112-120 |
| 20 | CUPVSiirt472 | 3 | 2.13 | 0.75 | 0.53 | 0.47 | 252-268 |
| 21 | CUPVSiirt476 | 2 | 1.28 | 0.25 | 0.22 | 0.19 | 154-157 |
| 22 | CUPVSiirt505 | 4 | 2.91 | 0.75 | 0.66 | 0.60 | 146-165 |
| 23 | CUPVSiirt509 | 4 | 3.20 | 1.00 | 0.69 | 0.63 | 171-188 |
| 24 | CUPVSiirt565 | 2 | 1.28 | 0.25 | 0.22 | 0.19 | 146-147 |
| 25 | CUPVSiirt569 | 3 | 2.57 | 0.33 | 0.61 | 0.54 | 95-102 |
| 26 | CUPVSiirt660 | 3 | 2.67 | 0.75 | 0.63 | 0.55 | 138-146 |
| 27 | CUPVSiirt719 | 2 | 1.88 | 0.25 | 0.47 | 0.36 | 190-198 |
| 28 | CUPVSiirt742 | 3 | 2.13 | 0.50 | 0.53 | 0.47 | 218-232 |
| 29 | CUPVSiirt743 | 3 | 2.00 | 0.33 | 0.50 | 0.45 | 159-173 |
| 30 | CUPVSiirt768 | 3 | 2.57 | 0.67 | 0.61 | 0.54 | 206-212 |
| 31 | CUPVSiirt782 | 4 | 4.00 | 1.00 | 0.75 | 0.70 | 180-202 |
| 32 | CUPVSiirt794 | 3 | 1.68 | 0.50 | 0.41 | 0.37 | 206-208 |
| 33 | CUPVSiirt796 | 2 | 1.60 | 0.50 | 0.38 | 0.30 | 106-112 |
| 34 | CUPVSiirt803 | 2 | 2.00 | 1.00 | 0.50 | 0.38 | 240-256 |
| 35 | CUPVSiirt836 | 4 | 3.56 | 0.25 | 0.72 | 0.67 | 144-168 |
| 36 | CUPVSiirt838 | 2 | 1.28 | 0.25 | 0.22 | 0.19 | 139-142 |
| 37 | CUPVSiirt847 | 2 | 1.28 | 0.25 | 0.22 | 0.19 | 249-253 |
| 38 | CUPVSiirt876 | 4 | 2.91 | 0.50 | 0.66 | 0.60 | 187-203 |
| 39 | CUPVSiirt956 | 3 | 2.67 | 0.00 | 0.63 | 0.55 | 131-135 |
| 40 | CUPVSiirt961 | 2 | 1.80 | 0.00 | 0.44 | 0.35 | 190-194 |
| 41 | CUPVSiirt986 | 3 | 2.46 | 0.75 | 0.59 | 0.51 | 146-153 |
| 42 | CUPVSiirt1003 | 2 | 1.80 | 0.67 | 0.44 | 0.35 | 81-97 |
| 43 | CUPVSiirt1008 | 2 | 1.80 | 0.00 | 0.44 | 0.35 | 163-165 |
| 44 | CUPVSiirt1017 | 2 | 1.60 | 0.50 | 0.38 | 0.30 | 230-232 |
| 45 | CUPVSiirt1021 | 3 | 2.46 | 1.00 | 0.59 | 0.51 | 127-135 |
| 46 | CUPVSiirt1043 | 3 | 2.00 | 0.67 | 0.50 | 0.45 | 113-157 |
| 47 | CUPVSiirt1047 | 2 | 2.00 | 1.00 | 0.50 | 0.38 | 133-144 |
| 48 | CUPVSiirt1053 | 2 | 2.00 | 1.00 | 0.50 | 0.38 | 145-153 |
| 49 | CUPVSiirt1055 | 2 | 1.80 | 0.67 | 0.44 | 0.35 | 175-180 |
| 50 | CUPVSiirt1062 | 3 | 2.57 | 1.00 | 0.61 | 0.54 | 146-151 |
| 51 | CUPVSiirt1071 | 2 | 1.38 | 0.33 | 0.28 | 0.24 | 126-135 |
| 52 | CUPVSiirt1120 | 2 | 1.60 | 0.00 | 0.38 | 0.30 | 193-196 |
| 53 | CUPVSiirt1145 | 4 | 3.60 | 0.67 | 0.72 | 0.67 | 150-168 |
| 54 | CUPVSiirt1153 | 2 | 1.88 | 0.75 | 0.47 | 0.36 | 172-194 |
| 55 | CUPVSiirt1182 | 2 | 1.60 | 0.00 | 0.38 | 0.30 | 159-171 |
| 56 | CUPVSiirt1191 | 2 | 1.28 | 0.25 | 0.22 | 0.19 | 154-164 |
| 57 | CUPVSiirt1202 | 3 | 2.13 | 0.75 | 0.53 | 0.47 | 180-194 |
| 58 | CUPVSiirt1224 | 2 | 1.28 | 0.25 | 0.22 | 0.19 | 268-273 |
| 59 | CUPVSiirt1238 | 4 | 3.56 | 0.75 | 0.72 | 0.67 | 229-252 |
| 60 | CUPVSiirt1243 | 2 | 1.60 | 0.00 | 0.38 | 0.30 | 134-136 |
| 61 | CUPVSiirt1260 | 2 | 1.80 | 0.00 | 0.44 | 0.35 | 151-157 |
| 62 | CUPVSiirt1278 | 2 | 1.60 | 0.00 | 0.38 | 0.30 | 217-220 |
| 63 | CUPVSiirt1326 | 2 | 1.88 | 0.25 | 0.47 | 0.36 | 186-188 |
| 64 | CUPVSiirt1345 | 3 | 2.57 | 0.33 | 0.61 | 0.54 | 173-191 |
| 65 | CUPVSiirt1372 | 2 | 1.80 | 0.00 | 0.44 | 0.35 | 123-130 |
| 66 | CUPVSiirt1400 | 3 | 2.91 | 0.75 | 0.66 | 0.58 | 169-197 |
| 67 | CUPVSiirt1402 | 3 | 2.13 | 0.50 | 0.53 | 0.47 | 209-213 |
| 68 | CUPVSiirt1405 | 4 | 3.56 | 0.25 | 0.72 | 0.67 | 191-223 |
| 69 | CUPVSiirt1413 | 2 | 1.38 | 0.33 | 0.28 | 0.24 | 184-196 |
| 70 | CUPVSiirt1438 | 2 | 1.38 | 0.33 | 0.28 | 0.24 | 266-286 |
| 71 | CUPVSiirt1457 | 2 | 1.28 | 0.25 | 0.22 | 0.19 | 156-157 |
| 72 | CUPVSiirt1477 | 2 | 1.88 | 0.25 | 0.47 | 0.36 | 118-120 |
| 73 | CUPVSiirt1547 | 2 | 1.38 | 0.33 | 0.28 | 0.24 | 120-126 |
| 74 | CUPVSiirt1567 | 2 | 2.00 | 1.00 | 0.50 | 0.38 | 189-203 |
| 75 | CUPVSiirt1611 | 2 | 1.80 | 0.00 | 0.44 | 0.35 | 193-194 |
| 76 | CUPVSiirt1652 | 2 | 1.60 | 0.00 | 0.38 | 0.30 | 167-172 |
| 77 | CUPVSiirt1655 | 3 | 2.46 | 0.50 | 0.59 | 0.51 | 170-184 |
| 78 | CUPVSiirt1667 | 2 | 1.60 | 0.50 | 0.38 | 0.30 | 149-152 |
| 79 | CUPVSiirt1734 | 2 | 2.00 | 0.50 | 0.50 | 0.38 | 178-185 |
| 80 | CUPVSiirt1749 | 2 | 1.28 | 0.25 | 0.22 | 0.19 | 133-143 |
| 81 | CUPVSiirt1759 | 3 | 2.57 | 0.67 | 0.61 | 0.54 | 147-153 |
| 82 | CUPVSiirt1764 | 4 | 3.20 | 0.50 | 0.69 | 0.63 | 155-176 |
| 83 | CUPVSiirt1797 | 6 | 4.57 | 1.00 | 0.78 | 0.75 | 159-177 |
|  | Total | 217 |  |  |  |  |  |
|  | Mean | 2.6 | 2.13 | 0.50 | 0.49 | 0.41 |  |
